# Exploring the binding pathways of the 14-3-3ζ protein: Structural and free-energy profiles revealed by Hamiltonian replica exchange molecular dynamics with distancefield distance restraints

**DOI:** 10.1371/journal.pone.0180633

**Published:** 2017-07-20

**Authors:** Gabor Nagy, Chris Oostenbrink, Jozef Hritz

**Affiliations:** 1 CEITEC-MU, Masaryk University, Brno, Czech Republic; 2 Institute for Molecular Modeling and Simulation, University of Natural Resources and Life Sciences, Vienna, Austria; Weizmann Institute of Science, ISRAEL

## Abstract

The 14-3-3 protein family performs regulatory functions in eukaryotic organisms by binding to a large number of phosphorylated protein partners. Whilst the binding mode of the phosphopeptides within the primary 14-3-3 binding site is well established based on the crystal structures of their complexes, little is known about the binding process itself. We present a computational study of the process by which phosphopeptides bind to the 14-3-3ζ protein. Applying a novel scheme combining Hamiltonian replica exchange molecular dynamics and distancefield restraints allowed us to map and compare the most likely phosphopeptide-binding pathways to the 14-3-3ζ protein. The most important structural changes to the protein and peptides involved in the binding process were identified. In order to bind phosphopeptides to the primary interaction site, the 14-3-3ζ adopted a newly found wide-opened conformation. Based on our findings we additionally propose a secondary interaction site on the inner surface of the 14-3-3ζ dimer, and a direct interference on the binding process by the flexible C-terminal tail. A minimalistic model was designed to allow for the efficient calculation of absolute binding affinities. Binding affinities calculated from the potential of mean force along the binding pathway are in line with the available experimental estimates for two of the studied systems.

## Introduction

14-3-3 proteins are important regulatory factors found in all kingdoms of life and are vital for the survival of higher organisms. In mammals, the 14-3-3 family consists of seven isoforms that can be found in large abundance within the brain. The human 14-3-3ζ isoform was selected for this project because of its high biological relevance. 14-3-3 proteins function mainly as dimers, which are composed of two 28-kDa monomers that are both capable of binding phosphorylated serine (pS) and threonine (pT) motifs in other proteins. Crystal structures of all seven mammalian homodimers are now available and show that each monomer is composed of nine α-helices, arranged in an antiparallel fashion. The helices form an amphipathic groove that mediates pS and pT target binding [1]. Most 14-3-3 targets have two phosphoserine/threonine-containing motifs with a consensus sequence RSXpSXP (mode I) or RX[FY]XpSXP (mode II) [2], representing the optimal recognition sites for 14-3-3. Upon binding to these sites 14-3-3 proteins induce conformational changes in their target protein, (and ‘finish the job’) when phosphorylation alone may lack the power to drive the necessary allosteric changes for modulating the activity of an intracellular protein. Owing to their dimeric nature, 14-3-3 proteins are capable of distinguishing between non-, single- and double- phosphorylated binding protein partners; in this sense 14-3-3 proteins are considered to act as coupled binary devices [3]. Recently, we have presented an approach based on experimental ^31^P NMR spectroscopy which revealed that a double-phosphorylated protein can be complexed with the 14-3-3ζ dimer in a much more dynamic fashion than was originally thought. In addition to the traditionally considered single partner with two phosphorylation sites occupying the individual binding cavities within the 14-3–3ζ dimer, two more major binding modes were confirmed [4]. All that is currently known about the structural features of the phosphopeptide binding to 14-3-3 proteins originates from the available crystal structures of 14-3-3 proteins in apo and holo state. Comparison of the various 14-3-3 crystal structures also revealed the different width of the main peptide binding groove, thus suggesting a dynamic opening process [5].

Here, we present the structural and energetic features of selected phosphopeptides along their binding/unbinding pathways to/from the 14-3-3ζ binding site, determined by enhanced sampling computational approaches. The main applied methodology is based on Hamiltonian replica exchange molecular dynamics (HRE-MD) combined with distancefield (DF) distance restraints [6], which has several advantages over the more conventional umbrella sampling and distance restraints approaches. HRE-MD is a highly parallel perturbed molecular dynamics (MD) technique, whereby each parallel simulation (replica) represents a discrete state along a thermodynamic pathway. The simulation of replicas are independent of each other, however, at given time periods the replicas can exchange their coordinates to allow the ensembles of each replica to include conformations derived from multiple starting coordinates. The application of HRE-MD instead of a set of individual MD simulations with different distance restraints significantly enhances sampling efficiency. HRE-MD could be combined with regular distance restraints, however, such an approach could lead to protein damage when coordinates at larger Cartesian distances switch to shorter distances [7]. Applying DF restraints avoids the protein damage whilst the ligand is pulled into the binding site by using an altered reaction coordinate, for which the distance to the binding site is defined by the shortest sterically possible pathway. Combining DF restraints and HRE-MD allows the simulation coordinates of the replicas to traverse reversibly between the various states of the binding pathway and represent conformations in structural ensembles restrained at different DF distances that would otherwise require much longer simulation times due to kinetic barriers. The set of thermodynamic ensembles that is generated is used to determine the structural features of the peptide-binding pathways as well as the corresponding potential-of-mean-force (PMF) profiles which can be used for the calculation of the absolute binding affinities.

## Results and discussion

We studied the binding of phosphorylated peptides to the 14-3-3ζ protein by constructing two different models of 14-3-3ζ, one containing a full length 14-3-3ζ dimer (dim) and one including only a truncated 14-3-3ζ monomer (tmon) without the flexible C-terminal stretch (tail, aa. 230–245). The proper simulation of a full length 14-3-3ζ dimer requires a large simulation box in order to avoid artificial periodic effects arising from the opening motion of 14-3-3ζ and the flexible C-terminal regions. Furthermore, the presence of the flexible C-terminal stretch also significantly slowed down the convergence of calculated free energies, as is demonstrated by the PMF calculations (see below). Four different phosphopeptide fragments are used as models for this study which were derived from the diphosphorylated PKC-ε and C-RAF kinase binding sites for 14-3-3ζ, and are referred to as the head and tail fragments of peptides 1 and 2, respectively (p1h, p1t, p2h, p2t), based on their location in the full protein sequence. These phosphopeptide fragments were chosen because their crystal structure bound to 14-3-3ζ was available and their binding affinities were previously measured [8–10].

**Fig 1:**
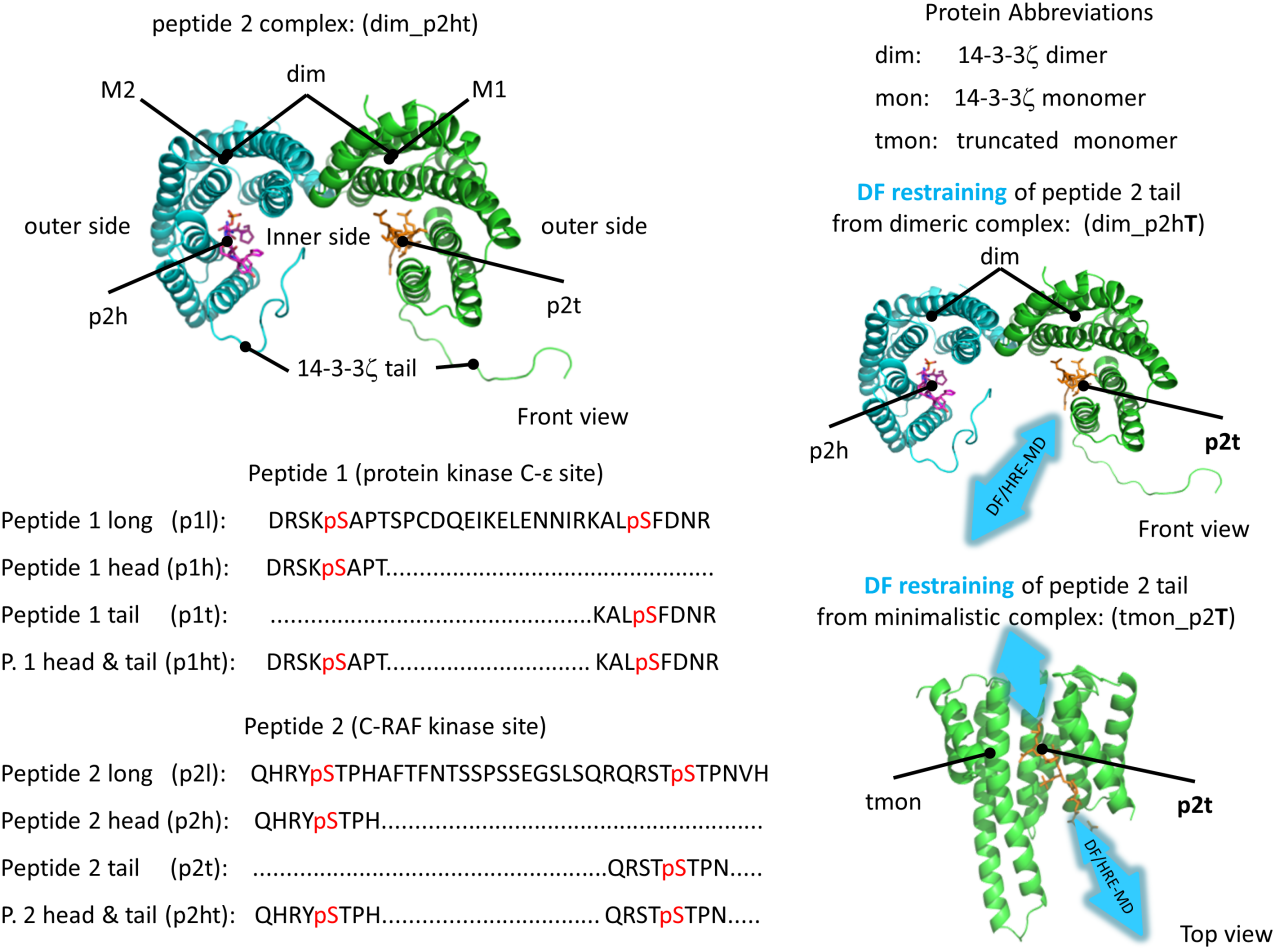
3D representation, nomenclature and sequence of the model molecules used during the simulations. Dimer and monomer 14-3-3ζ systems (on the right) were simulated with fragments of phosphopeptide 1 and 2 (sequence shown on the left). The phosphoserine in the sequence is highlighted in red. 14-3-3ζ monomers are represented as cartoons in green and cyan, phosphopeptides are shown in stick representations in orange and purple. The phosphopeptide under DF restraining is labelled by an upper-case letter in the abbreviations.

Phosphopeptide sequences, abbreviations and naming conventions used in this work are given in Fig 1. The monomers of 14-3-3 proteins are horse-shoe shaped, and the first four α-helices form the dimerization interface. The two monomers of a 14-3-3ζ dimer are denoted as M1 and M2 when a distinction is necessary. The helices three, five, seven and nine of each monomer form the phosphopeptide binding grooves on the inner side of the 14-3-3ζ dimer. When referring to our simulations we denote our simulated complex by first defining the 14-3-3ζ model (dim or tmon) followed by peptide fragment present in the simulation (either p1h, p1t, p2h, p2t or nothing, in case of apo simulations). In the case of dimeric simulations, if two peptide fragments are present we will denote it by p1ht or p2ht representing either peptide 1 (region 342–372 of protein kinase C-ε) or peptide 2 (region 229–264 of C-RAF kinase) fragments. In addition, when DF distance restraints are applied to pull one of the phosphopeptide fragments from its respective binding groove, we mark this fragment with an upper-case letter (e.g. tmon_p2T or dim_p2hT).

Our results are divided into three sections. The first section describes phosphopeptide binding pathways elucidated from the DF/HRE-MD simulations. We characterized the binding/unbinding pathways of studied 14-3-3ζ/phosphopeptide complexes by determining the most important protein-peptide interactions between 14-3-3 and its binding partner, and identifying the regions most frequently populated by the restrained phosphopeptide. In the second section we focus on the structural changes along the binding pathways for both 14-3-3ζ and the phosphopeptide. Here we aim to identify the large-scale protein motions, which may be important for the phosphopeptide and protein binding. The third section presents the free energy changes along the binding pathways. The determined free-energy profiles allowed us to calculate estimates for the corresponding phosphopeptide binding affinities.

### 1. Major binding pathways of the 14-3-3ζ protein

In this section, we have elucidated the phosphopeptide binding pathways of 14-3-3ζ, using DF/HRE-MD simulations. We achieved this by gradually pulling one of the phosphopeptides bound to the 14-3-3ζ from its respective binding site in a reversible HRE-MD process. During the DF/HRE-MD simulations replicas with increasing index numbers restrained the phosphopeptide to regions with DF distances further away from the peptide binding groove (interaction site 1, IS1). Note that for every dimeric 14-3-3ζ complexed with two phosphopeptide fragments (e.g. p1h and p1t in dim_p1Ht) one phosphopeptide fragment (p1h in case of dim_p1Ht) per process was pulled from its monomer, whilst the other (p1t in case of dim_p1Ht) remained in its respective monomer binding site (Fig 1). Such a setup avoids complications arising from the two available binding sites (for p1h) within one 14-3-3ζ dimer.

#### 1.1. Characterization of the phosphopeptide binding pathways

**Fig 2:**
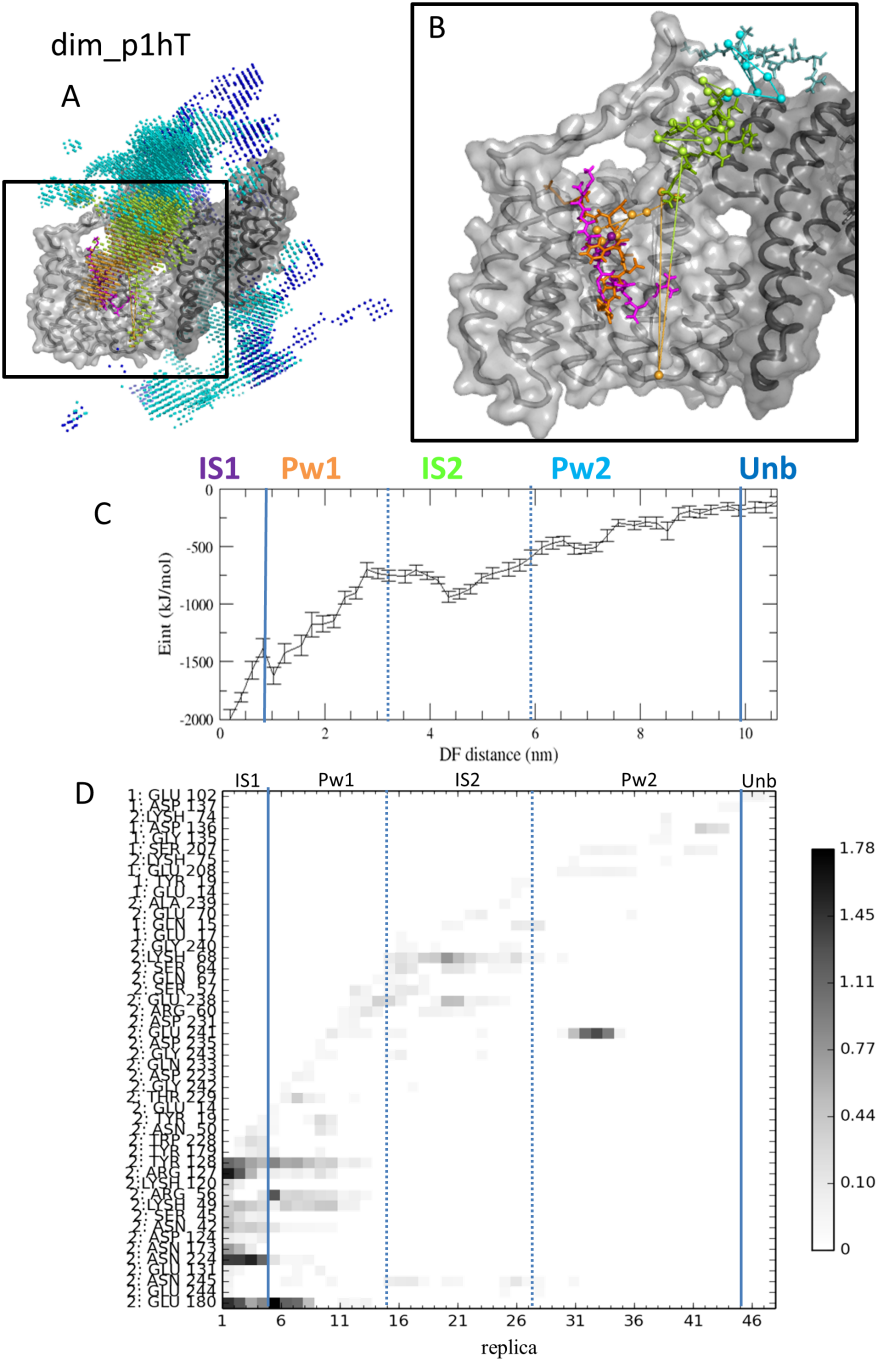
Pathway visualization of the DF/HRE-MD simulation dim_p1hT. A) The volume sampled by the p1t phosphopeptide during the simulation is shown by dots around the 14-3-3ζ protein coloured based on their position along the pathway. The most probable points in space to find the peptide in for each replica (probability density peaks) are represented by larger spheres. B) Density peaks and a few representative structures corresponding to the density peaks, coloured according to their position along the pathway. Replica density peaks are connected by lines to visualize the binding pathway. The 14-3-3ζ protein in panels A-B is shown as a surface representation, with the two monomers shown in light and dark grey. C) Average interaction energy between 14-3-3ζ and p1t. D) Interaction map between any atom of the pulled p1t peptide and the amino acids of the 14-3-3ζ protein, summarized for each replica (only amino acids with at least 0.1 hydrogen bond/salt bridge on average are shown). The scale indicates the average number of interactions.

In order to characterize the phosphopeptide binding pathways of 14-3-3ζ, we monitored the position and interactions of the phosphopeptide during its binding/unbinding process. Fig 2 summarizes the results of such an analysis for simulation dim_p1hT (S1–S7 Figs corresponds to the other 7 DF/HRE-MD simulations). The position of the DF-restrained phosphopeptide was tracked on a three dimensional grid through its virtual atom (as described in Methods section) near the pSer residue during the DF/HRE-MD simulations (Fig 2A). The most occupied/preferred positions of the phosphopeptide at various DF distances were aligned along a dominant binding/unbinding pathway (as shown in Fig 2B). Similar dominant pathways were observed for seven out eight simulations (as shown in S1B–S7B Figs).

The analysis of phosphopeptide-protein interaction energies (Fig 2C) shows a local minimum around the DF distance of 3.5–6.0 nm, in addition to the global minimum of original binding site at the DF distance of about 0.2 nm. The local interaction energy minimum co-occurred with stable, specific interactions with certain 14-3-3ζ residues around amino acids 64–70 (Fig 2D). Similar characteristic interaction energy minima were also observed for all seven simulations with a dominant pathway, including stable interactions between the phosphopeptide and the same 14-3-3ζ residues (64–70, in S1D–S6D Figs), suggesting a previously unknown secondary interaction site. To better characterize our binding pathways, we divided the pathways into five parts; starting from the primary interaction site (IS1) within the main phosphopeptide binding groove, followed by the early pathway (Pw1) between the two interaction sites, the vicinity of the secondary interaction site (IS2), the late pathway (Pw2) after IS2, and the unbound state (Unb), where the pathway becomes diffuse and no significant interactions with 14-3-3ζ are observed for the phosphopeptide.

Experimental support for the existence of the IS2 site can possibly be found in previously published ^31^P NMR titration data of the doubly phosphorylated (at positions 19 and 40) human tyrosine hydroxylase 1 peptide binding to 14-3-3ζ [4]. Fig 2A of the referred article shows an “unexpected” peak labelled as pS40*, which was different from pS40 in the free state or bound to IS1. One of the possible explanations is that while pS19 is bound to IS1, pS40 can interact with IS2—still being partially solvent exposed—resulting in the additional peak.

#### 1.2. Comparison between phosphopeptide pathways

**Fig 3:**
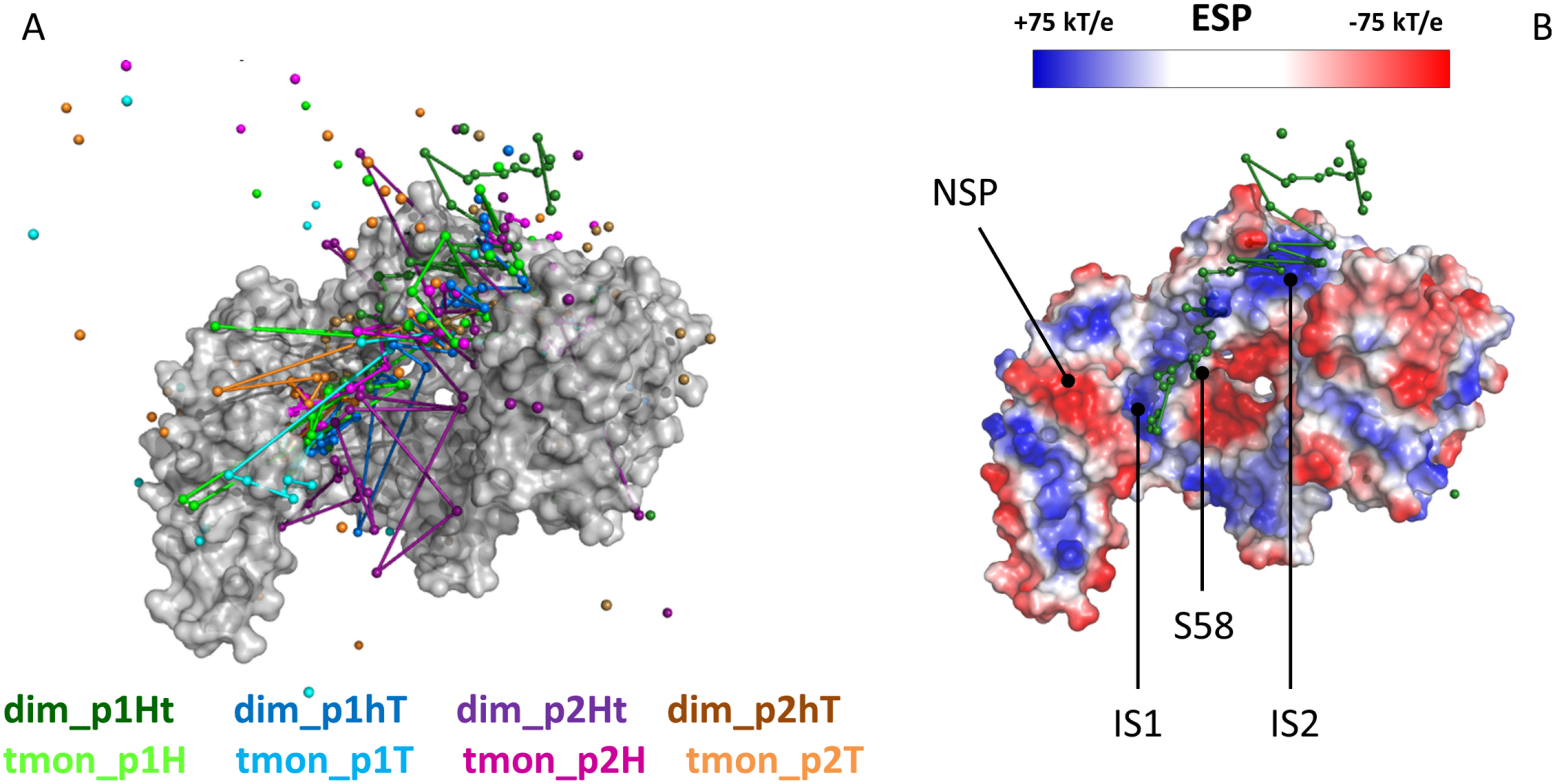
Comparison of binding pathways of different phosphopeptides. A) Binding pathways from DF/HRE-MD simulations depicted as connected dots referring to the probability density peaks along the respective pathway (See Table 1 for more information). B) Electrostatic surface potential (ESP) of 14-3-3ζ in blue, white and red for positive, neutral, and negative surface patches, respectively. The positively charged main interaction site (IS1), and secondary interaction site (IS2) are connected by a positive surface along the binding pathways. A negative surface patch (NSP) involved in the binding process is also indicated. Serine58 (S58), a phosphorylation site is located near the binding pathway. For clarity, the 14-3-3ζ C-terminal tail is not shown.

Comparing the binding pathways obtained for the four studied phosphopeptide fragments (with both 14-3-3ζ models), we found a remarkable similarity between their most probable pathways in 7 out of 8 simulations (with the exception of dim_p2Ht, Fig 3A). The dominant pathways generally followed helix three, “sliding” from IS1 in the primary binding groove to IS2, and becoming increasingly diffuse before the final detachment of phosphopeptide and reaching the Unb state. This general behaviour indicates the existence of one dominant pathway for all four phosphopeptides, observed both in the dimeric and truncated monomeric simulations. Examining the electrostatic surface potential (ESP) in multiple 14-3-3ζ conformations showed both IS1 and IS2 display highly positive surface potentials; and the connecting region along helix three also exhibits a mildly positive surface potential (Fig 3B). Thus, these areas can form favourable electrostatic interactions with a negatively charged phosphoserine side chain, which could explain the presence of a dominant binding pathway.

In addition, based on the ESP we identified a negative surface patch (NSP) which was often found to accommodate the positively charged side chain in position -3 or -2 relative to the phosphorylation site of the peptide fragment. The NSP is primarily composed of residues Y179, E180, N183, D223, N224, L227 and W228. The NSP provides an additional anchor point on the binding pathway and also concurs with the strong selection of positive amino acids in this position as reported by Jaffe *et*. *al*. [2].

It was previously reported [11], that phosphorylation and mutation of S58 into negative amino acids lead to dimer dissociation and a (usually negative) change in the 14-3-3 binding affinity, independently of the oligomeric state. S58 is located near the dimer interface of 14-3-3ζ and directly next to the binding pathway between IS1 and IS2. A negatively charged residue in position 58 could negate the mildly positive surface potential (Fig 3 and S13 Fig), and disrupt the observed binding pathway, which is in line with the observed experimental changes.

**Fig 4:**
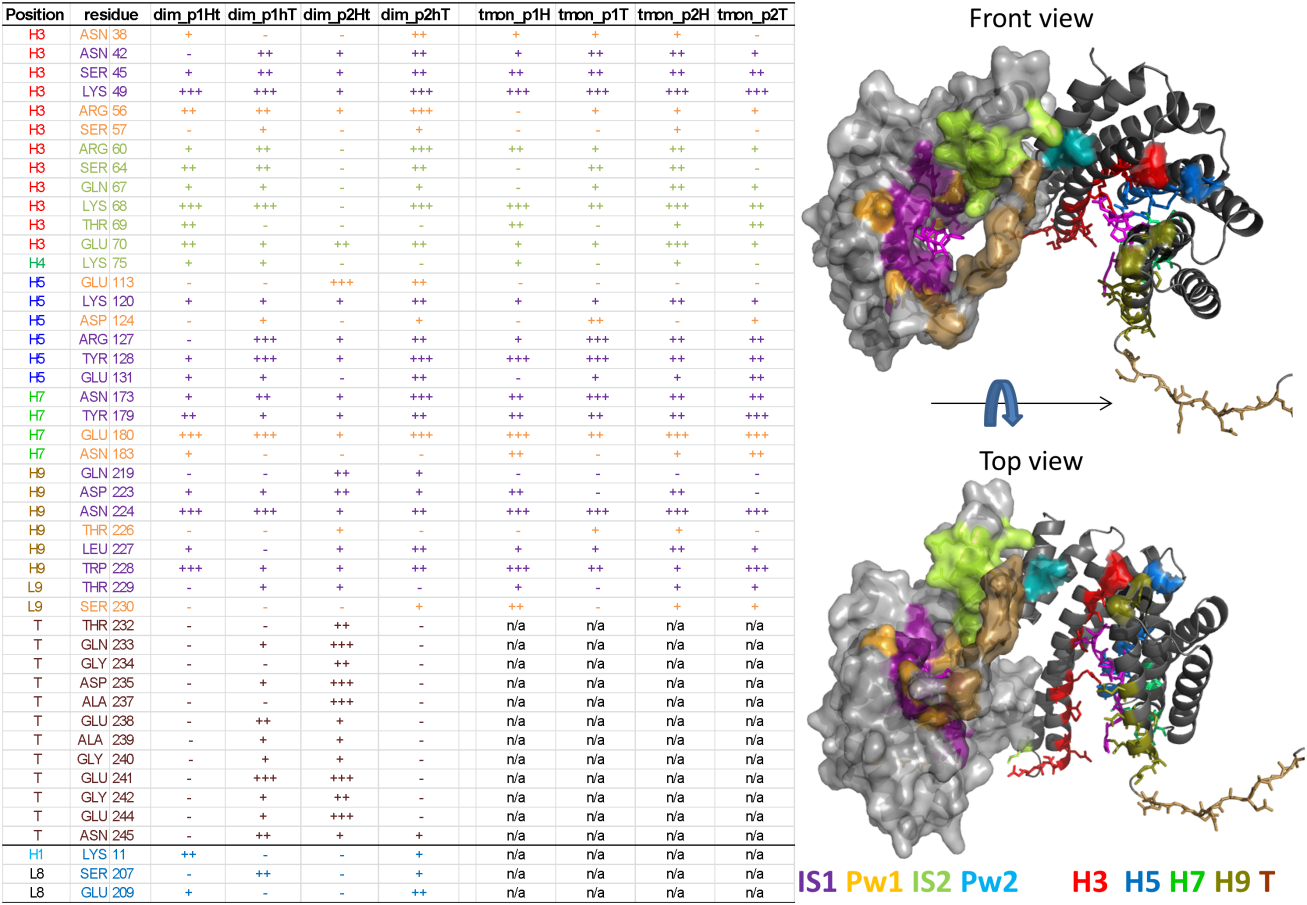
14-3-3ζ residues involved in the phosphopeptide interactions. The table on the left side shows the 14-3-3ζ amino acids, which were found important based on the interaction map analysis (Fig 2, S1–S7 Figs) of the DF/HRE-MD simulations. Entries in the various simulations are marked as not applicable (n/a), not significant (-), significant (+), important (++), or major interaction partners (+++). The last three amino acids in the table were interaction partners from the other monomer. The right side shows the three dimensional structure of the 14-3-3ζ protein, where the monomers are depicted in cartoon and surface representations, respectively. The amino acids are depicted in stick representation, coloured differently for the two monomers. In the cartoon representation, peptide-interacting amino-acids are coloured according their position in the secondary structure, where helices 3, 5, 7 and 9 are shown in red, blue, green and brown, respectively, while the C-terminal stretch is depicted in light purple. For the surface representation, amino acids found significant only in the bound-state replicas (IS1) are shown as dark purple, amino acids along the binding pathway are marked in orange, light green and light blue, if they appeared prior, in, or after the secondary interaction site (IS2). See S8 Fig for details.

We identified the amino acids of 14-3-3ζ which were important phosphopeptide interaction partners at each of the five stages of the binding pathway. Most interactions between 14-3-3ζ and the phosphopeptides were polar hydrogen bonds or salt bridges. During our simulations, as the phosphopeptide sampled the available volumes along the dominant pathway, the overall probability to find a particular 14-3-3 amino acid interacting with the restrained phosphopeptide was calculated (shown in S8 Fig) as function of the replica number (restraining DF distance). In Fig 4 we display the table of all the 14-3-3ζ residues found significant (over 10% probability at a given replica) in this analysis. The residues are coloured according to the stage in which the residue was found most significant.

In dark purple we have depicted residues found most significant while the phosphopeptide is bound in IS1 including many residues previously identified as crucial interaction partners (such as Lys 49, and Arg 127) based on 14-3-3 crystal structures, and residues from the negative surface patch such as Asp 224, which mostly interacted with the positively charged residues of the phosphopeptide. The second group of amino acids (shown in orange) were found most prominent on the early pathway (Pw1) between IS1 and IS2. Many of these residues were probable interaction partners already when the peptide was in IS1 (such as Glu 180 and Arg 56), whilst other residues became significant only as the phosphopeptides left the primary binding site (e.g. Glu 113 and Asp 124).

The third group of residues were most prominent whilst the phosphopeptide visited IS2. Most of these residues (e.g. Ser 64, Lys 68 and Lys 75) are located in the positive surface patch at the end of helix three (marked with light green in Fig 4), near the dimerization interface. There were also three residues (Lys 11, Ser 207 and Glu 209, shown light blue) considered as important interaction partners only during our dimeric DF/HRE-MD simulations. In dimeric simulations these residues from the other, 14-3-3 monomer enhanced and expanded the positive surface patch at IS2, and prevented the phosphopeptide to diverge from a dominant pathway for ~1.0 nm longer in the distance field during the late pathway stage (Pw2) compared to monomeric simulations.

The final group of interacting residues, were located on the disordered C-terminal region (aa. 231–245) of the monomer from which the peptide is being pulled, shown in brown in Fig 4. The bulk of the tail interactions were observed in the PW1, IS2 and Pw2 stage of the binding pathway (including the salt bridges with Glu 241 and Glu 244), although some of the tail residues could interact with the phosphopeptide fragments while they were bound to IS1 (Thr 232, Gly 234 and Ala 237, most prominently with the p2h).

Fig 4 also highlights that the occurrence of phosphopeptide—tail interactions was very high in two of the DF/HRE-MD simulations (dim_p1hT and dim_p2Ht) and very low in the other two cases (dim_p1hT and dim_p2hT). The strongest interactions with the 14-3-3 tail region were observed for the simulation dim_p2Ht, which at the same time had the weakest interactions with IS2 and did not follow the dominant phosphopeptide binding pathway observed during the other seven DF/HRE-MD simulations (S7 Fig). These observations suggest that C-terminal tail may disrupt the dominant peptide binding pathway.

#### 1.3. Comparing the pathways within 14-3-3ζ dimeric and truncated monomeric model

The summary of our DF/HRE-MD simulations indicates a dominant phosphopeptide binding pathway for 14-3-3ζ where the phosphopeptide in the process of unbinding “slides” from the IS1 towards IS2 along the described pathway shown in Figs 3 and 4. Here, we summarize the differences observed between our full dimeric systems (dim) and the minimalistic, truncated, monomeric systems (tmon) during the detailed analysis of preferred positions and phosphopeptide interactions (shown in Figs 2–4 and S1–S8 Figs).Our minimalistic systems lacked the neighbouring monomer and the C-terminal region of the 14-3-3ζ protein, which, in the case of full dimeric systems, restricted the accessible space for the peptides and necessitated longer DF distances to reach the unbound state.

The truncated monomer pathways were more similar to each other near the main peptide binding groove (IS1), and showed enhanced interactions with helices 7–9, which were partially blocked by the C-terminal region in the dimers. The monomeric pathways fanned out after leaving the IS2 around 5 nm in the DF and sampled the outer side of 14-3-3ζ monomer before final detachment from the 14-3-3ζ surface, and showed no significant interactions above 7 nm.

Full dimeric simulations lead to a more complete picture of 14-3-3ζ binding pathways and could yield additional, biologically relevant information. The pathway comparison revealed a more localized phosphopeptide presence for all dimeric systems near the dimerization interface, along with new interaction partners from the other monomer (K11, S207, and E209). During dimeric HRE-MD simulations which followed the dominant pathway, phosphopeptides occupied the secondary interaction site (IS2) for a wider range of distances than their monomeric counterparts and had non-negligible interactions between the restrained peptide and the 14-3-3ζ dimer for DF distances smaller than 9.5 nm.

The interactions between the phosphopeptide and the C-terminal tail of the restrained 14-3-3 monomer showed a large variation between the four full dimeric simulations. In case of the p2h peptide fragment, strong interactions with the tail seemed to prevent the phosphopeptide to follow the dominant pathway (S7 Fig), which is a major difference compared to the truncated simulation with the same peptide (S2 Fig). The signs of direct interference from the C-terminal tail, and the tail-associated discrepancies in the full dimeric phosphopeptide binding simulations necessitated further investigation, shown below.

### 2. Structural changes along the binding pathway

The structural ensembles generated at different replicas within the DF/HRE-MD allowed us to analyse structural changes as function of the DF distance from IS1. The results of this analysis allowed us to monitor the slow conformational degrees of freedom which may affect the binding/unbinding process of 14-3-3ζ. In the following section we identify these large scale motions, and compare our DF/HRE-MD simulations in the bound (IS1) and unbound (Unb) states with unrestrained MD simulations under the same conditions and—whenever possible—experimental observations. This analysis allowed us to explore the observed discrepancies, assess the effect of these large scale motions on the phosphopeptide binding process during the limited sampling of our simulations, as well as effects of the applied DF/HRE-MD restraints on the system.

#### 2.1. The C-terminal region

**Fig 5:**
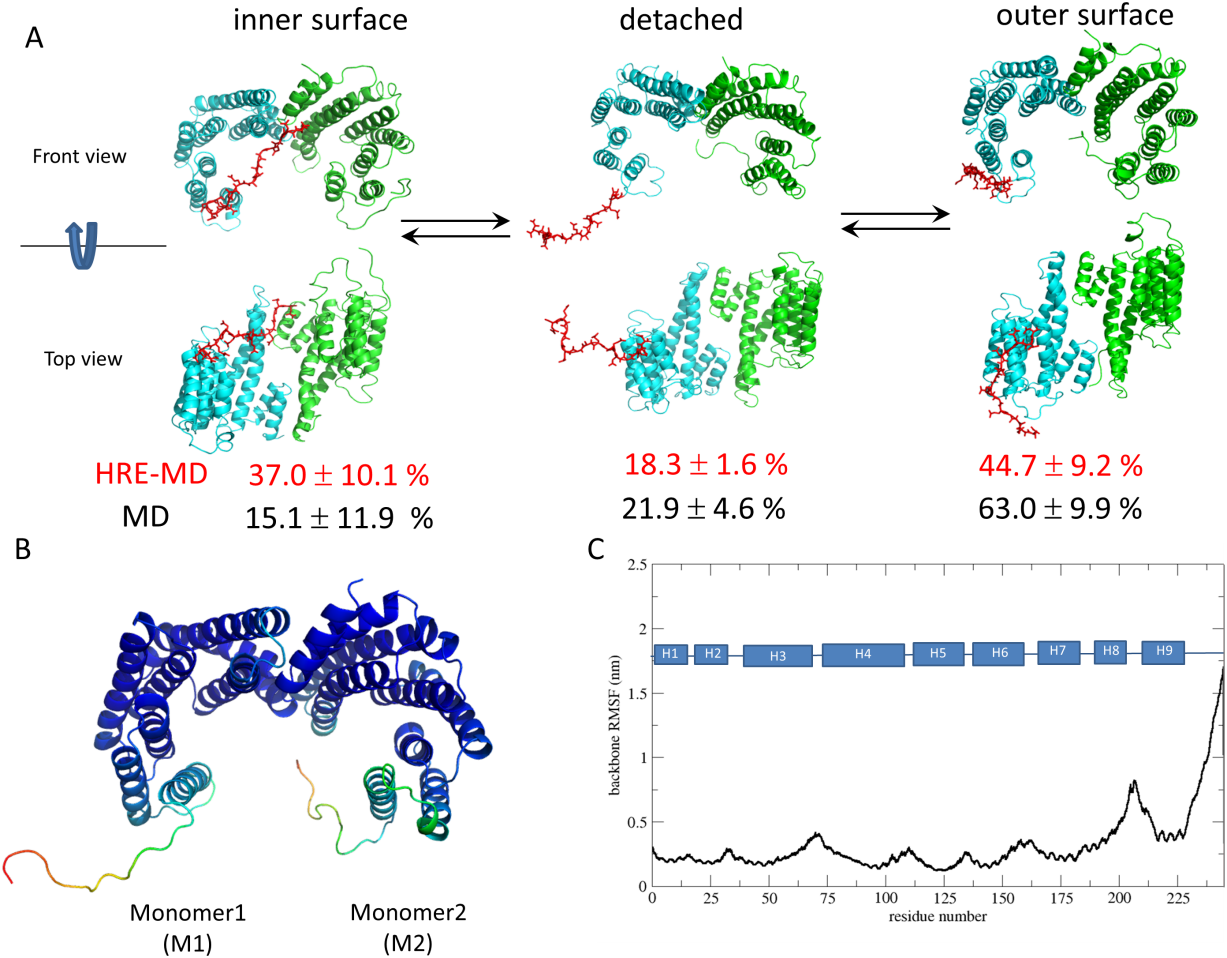
Flexibility of the 14-3-3ζ C-terminal tail. A) Representative structures of the conformational sub-states of the C-terminal stretch (marked in red) interacting with the inner or outer surface of 14-3-3ζ or being detached and exposed to the solvent. Populations of these conformational states as obtained from DF/HRE-MD (in red) and MD (in black) simulations are listed under the cartoon figures. B) 14-3-3 model starting conformation, coloured according to the backbone RSMF, where blue and red represent the least and most flexible amino-acids, respectively. C) The atom-positional root-mean-square fluctuations (RMSF) of the 14-3-3ζ backbone atoms.

The 14-3-3ζ proteins contain a 15 amino acid long C-terminal region, which is a highly flexible disordered segment of the protein (Fig 5). In our simulations two types of models were employed: full length dimeric (dim) models, and truncated monomeric (tmon) models lacking the C-terminal tail. The C-terminal regions in our full-length models visited both the inner (dimer interface formed by helices 3–4 of both monomers) and outer (helices 7–9) side of the 14-3-3ζ protein surface, as well as free conformations in which the tail was detached from the protein surface (Fig 5A). The position of the C-terminal tail in the simulations was determined based on the distances measured between the terminal carbonyl (of N245) and the Nζ atom of K68 (in IS2), Nζ of K120 (in IS1), and Cγ of D197 (on the outer side), with cut-off distances 2.5, 2.0, and 2.5 nm, respectively. The tail was considered to be on the inner side of 14-3-3ζ if the terminus was within the cut-off distance of IS1 or IS2, considered to be on the outer side if it was within the cut-off distance of D197 but not the other two residues, and was considered free otherwise. The detailed population distributions and number of transitions between the four states are shown in S2 and S3 Tables for HRE-MD and MD simulations, respectively.

The exchange between the three conformational sub-states of the protein tail was a process observed, but not properly sampled on the time scales of our DF/HRE-MD simulations. Analysis of the tail residence during the DF/HRE-MD simulations (S2 Table) revealed a strong bias towards the conformation of the tail at the start of the DF/HRE-MD run (shown in Fig 5B). The tail of monomer 1 (M1) at the start of the simulations was facing outwards, while the tail of monomer 2 (M2) was facing towards the inner side of the 14-3-3 dimer (in both cases the starting conformation was detached from the surface). During the simulations each C-terminal region of M1 dominantly sampled free conformations and the outer surface of 14-3-3ζ, while tail of M2 sampled free conformations and the inner surface. A few transitions from the inner to the outer side were observed for the M2 tail, suggesting a slow conformational transition and preference towards the outer surface.

The bias of the preferred C-terminal tail conformation also explains the differences observed in the phosphopeptide interaction analysis. During the simulations dim_p1Ht and dim_p2hT the peptide fragment was pulled from M1, and since the C-terminal tail was mostly located on the outer side of the 14-3-3 dimer, no or very little interaction between the tail and the phosphopeptide was observed (Fig 4). On the other hand, for the simulations dim_p1hT and dim_p2Ht the peptide was pulled from M2 and the tail was close to the inner surface and the phosphopeptide binding pathway, allowing for strong tail-peptide interactions.

The explanation for the difference between the p2h binding pathways in tmon_p2H (S2 Fig) and dim_p2Ht (S7 Fig) is that the C-terminal tail partially buried and neutralized the positive surface potential of IS2 in dim_p2Ht. This prevented the p2h fragment in dim_p2Ht from following the dominant pathway, which consequently explored less likely, alternative binding/unbinding pathways. In the case of dim_p1hT (Fig 2), IS2 was more exposed, and the C-terminal tail formed salt bridges and remained attached to the phosphopeptide as it traversed through the dominant pathway (until the p1t fragment was pulled out of reach).

We compared the tail behaviour of the DF/HRE-MD simulations to four unrestrained MD simulations (two with and two without peptides) of 40 ns length (S3 Table). The unrestrained simulations showed a similar behaviour (black and red percentages in Fig 5A) as the DF/HRE-MD simulations. The tails showed a strong bias towards either the inner or outer side of the 14-3-3ζ dimer, and even fewer transitions between the two were observed. However once the C-terminal tail travelled from the inner surface to outer surface, we did not observe its return. Consequently the classical MD simulations have shown an even stronger preference towards the tail to be found near the outer surface. Despite this preference, the C-terminal tails in both DF/HRE-MD and unrestrained MD simulations remained the most flexible part of 14-3-3ζ (as shown in Fig 5C) with regular transitions between detached and surface bound conformations.

The biological function of the 14-3-3 C-terminal region is not fully understood. It was suggested previously [12] that the 14-3-3ζ C-terminal tail can have an auto-inhibitory effect by binding to the phosphopeptide binding groove of its respective 14-3-3ζ monomer (IS1). The C-terminal tail was not observed occupying the IS1 in any of our MD simulations, but this occurred transiently (<5) % in the HRE-MD simulations of dim complexes. Our results suggest that instead of occupying IS1, the tail rather interacts with amino acids on the outer surface of its 14-3-3 monomer, or amino acids from IS2. In addition, the C-terminal region also spent a considerable amount of time (~20%) being detached from the globular parts of 14-3-3ζ, and in some of the DF/HRE-MD simulations directly interacted with the phosphopeptides themselves during the binding process. Even though the sampling of the conformational sub-states is incomplete, our simulations are more in line with NMR studies that showed high flexibility and only transient interactions between the structured part of 14-3-3ζ and the C-terminal region [13].

#### 2.2. 14-3-3ζ monomer opening

**Fig 6:**
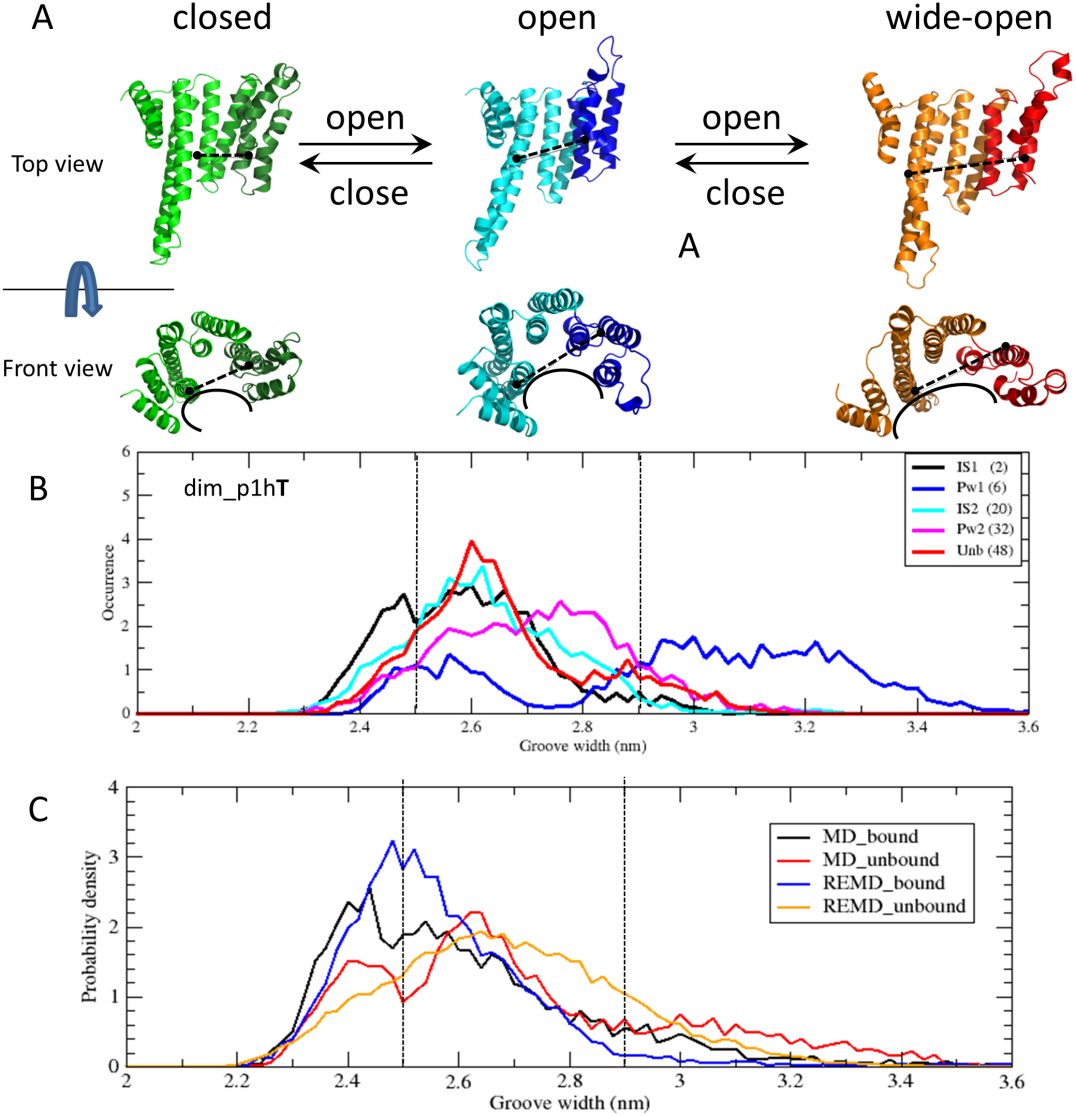
Changes in the 14-3-3ζ monomer groove width. A) Models of 14-3-3ζ at different levels of opening (the green, blue and orange colours mark closed, open and wide-open states, respectively), the last 3 helices are marked with a darker colour. B) Representative replicas of five different stages along the binding pathway (with the replica ID number shown in brackets) from DF/HRE-MD simulation dim_p1hT. The panel shows groove width distributions whilst the phosphopeptide moves from the binding site (IS1) to the unbound state (Unb). C) Average groove width distributions of 14-3-3ζ monomers as obtained from DF/HRE-MD bound (IS1) and unbound (Unb) states, and unrestrained MD simulations in holo (bound) and apo (unbound) states.

Structural changes in the 14-3-3ζ protein monomers leading to an opening or closing of the peptide-binding groove were previously reported using both computational and experimental methods [5,14]. This breathing motion was observed in our simulations as well, for both dim and tmon systems and their phosphopeptide complexes. We measured the groove width of a 14-3-3ζ monomer associated with the opening process, as the distance between the Cα atom of G53 (middle of helix 3) and L191 (middle of helix 8) (Fig 6A). Based on the measurement of the groove width distributions, we defined three sub-states (closed, open, wide-open) of the 14-3-3ζ monomer, depicted in Fig 6. We considered the 14-3-3ζ monomer closed, if the groove width was below 2.5 nm, wide-open above 2.9 nm and open in between.

We monitored the groove width distribution as the function of phosphopeptide location along the binding/unbinding pathway and found that the breathing motion in the restrained monomer was changed for all DF/HRE-MD simulations. Fig 6B presents the distribution of the groove widths for replicas representing the five stages along the binding pathway of the simulation dim_p1hT (replicas 2, 6, 20, 32 and 48 for IS1, Pw1, IS2, Pw2 and Unb, respectively). When the phosphopeptide was further away from the primary binding site (in the IS2, Pw2 or Unb stage) the groove width distributions were similar, centered on the open state (~2.6 nm), with a smaller probability to visit both the closed and wide-open states. When the phosphopeptide was bound to IS1, however, the interactions with residues from helices three, five and seven resulted in narrower groove width distributions which were also shifted towards the closed state. A similar observation was reported by Hu *et*. *al*. [14] (See S13 Fig). Interestingly, shortly after the phosphopeptide left IS1 (typically between replicas 6–11, Pw1) very wide grove width distributions were observed, with a high probability of the monomer from which the peptide was pulled adopting a wide-open conformation. The wide-open conformation in Pw1 is present for all our 14-3-3ζ/phosphopeptide models and is not dominant for any other part of the binding/unbinding pathway. These results (to our knowledge not reported before) indicate that the wide-open conformation of 14-3-3ζ may be necessary for the phosphopeptide ligand to detach from or enter into the primary binding site at IS1.

The distributions of the groove width for apo (unbound) and holo (bound) 14-3-3ζ in unrestrained MD simulations were also compared in Fig 6C with average groove width distributions for IS1 (bound) and Unb (unbound) DF/HRE-MD replicas. As the figure shows, the range of groove width distributions for the DF/HRE-MD and unrestrained MD simulations were very similar, and a shift towards the closed state was also observed between the bound and unbound monomers. The similarity between groove width distributions suggest, that the presence of a phosphopeptide strongly affected the breathing motion of 14-3-3ζ monomers in our simulations, but the applied DF restraints did not perturb this conformational degree of freedom directly.

#### 2.3. Inter-monomer twist within 14-3-3ζ homo-dimer

**Fig 7:**
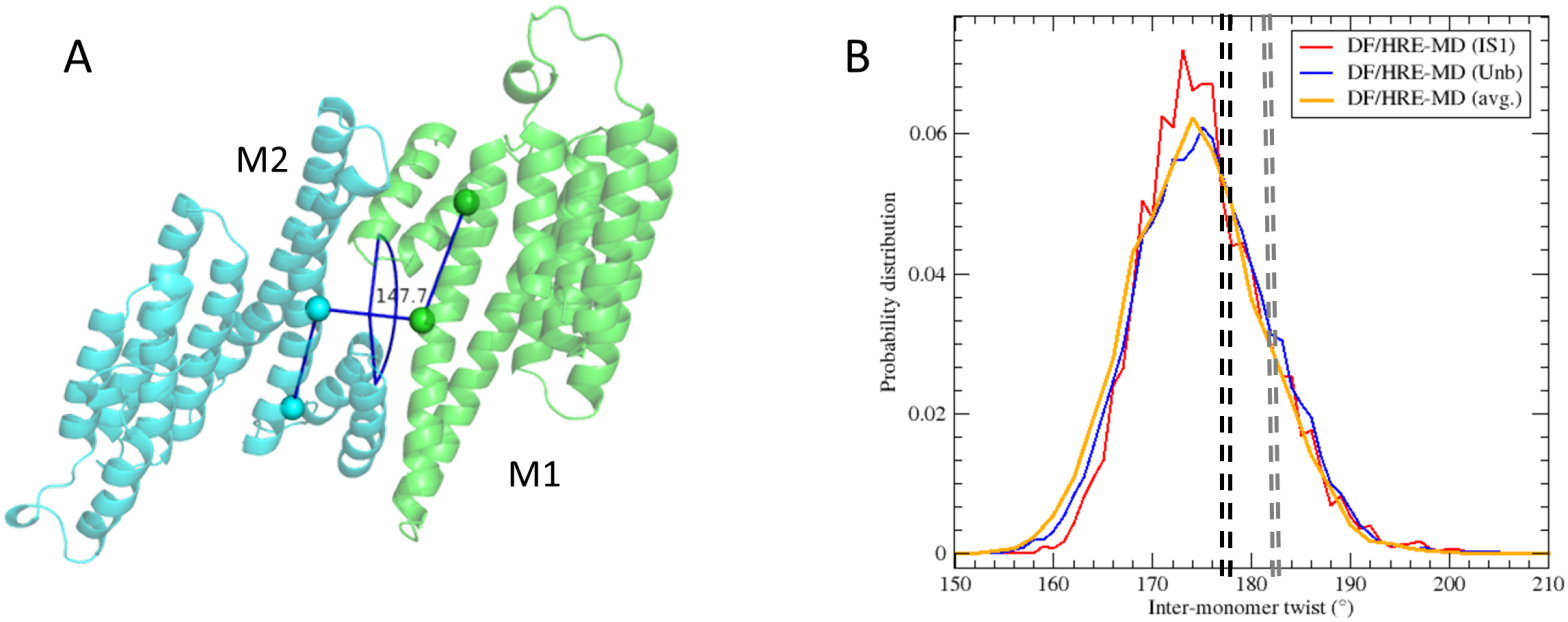
14-3-3ζ inter-monomer twist. A) The top view of the protein dimer, with the twist angle between the monomers displayed in dark blue. B) Probability distribution of inter-monomers twist angles averaged over all DF/HRE-MD simulations in the IS1 (bound) and Unb (unbound) stages, and for the whole pathway.

Apart from the internal motion of the monomers, motion of monomers relative to each other was also observed in our (dim) simulations. This motion was monitored by measuring the dihedral angle of the Cα atoms of the residues L43(M1)-A54(M1)-A54(M2)-L43(M2) where M1 and M2 indicate different monomers within the 14-3-3ζ dimer (Fig 7A).

During DF/HRE-MD simulations an inter-monomer twist of 175 ± 15° was observed, with little variation with respect to the pulled phosphopeptide fragment or its DF restraint position (shown in Fig 7B). The similar distributions of the inter-monomer twist angles suggest that this conformational degree of freedom is not involved in the phosphopeptide binding process. We speculate the inter-monomers twist may still be important for protein-protein binding processes, as it can help 14-3-3 to better adapt the binding interface.

In some preliminary regular MD simulations at lower salt content, the inter-monomers twist angle was significantly reduced (see Methods section and S9 Fig). However, the inter-monomer twist angles in the DF/HRE-MD simulations were consistent with the twist angles observed in the available crystal structures of 14-3-3ζ dimers (~180 ± 3° calculated from the pdb entries 2WHO and 1A4O) and yielded a better agreement with SAXS measurements (S14 Fig).

#### 2.4. Secondary structure changes of phosphopeptides along the binding pathway

**Fig 8:**
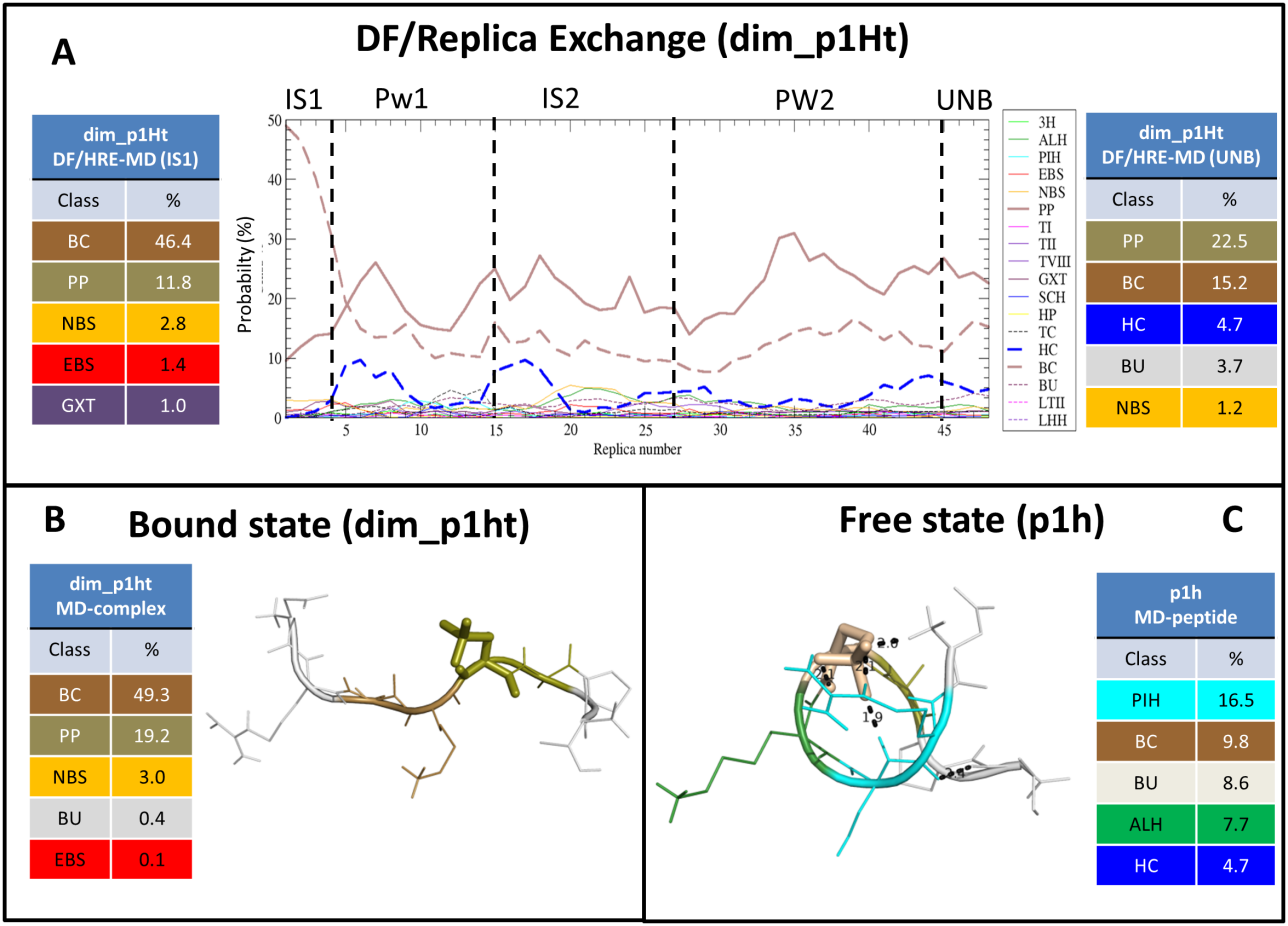
Phosphopeptide p1h secondary structure. Changes in the backbone structure are shown during DF/HRE-MD simulation dim_p1Ht (panel A) and unrestrained MD (panels B and C) simulations, analysed by the DISICL algorithm. The change in the average secondary structure content during the DF/HRE-MD simulation is shown in the middle of panel A, whilst the most dominant conformations in bound and unbound states are tabulated on the left and right side, respectively. Representative conformations for the bound and unbound states are depicted on the left and right sides, respectively, where the residues are coloured according to secondary structure classification. Intra-molecular hydrogen bonds are depicted as dashed lines. The tables besides the depictions show the 5 most populated secondary structure classes for the corresponding MD simulation and the appropriate (bound(IS1)/unbound) stage of the DF/HRE-MD simulation, respectively. The most populated DISICL classes are depicted in the following colours: π-helix (PIH)–cyan, Extended β-strand (EBS)–red, normal β-strand (NBS)–orange, polyproline-like (PP)–brown, turn type VIII (TVIII)–indigo, Gamma turn (GXT)–maroon, β-cap (BC)–gold, helix-cap (HC)–blue, turn-cap (TC)–black. DISICL secondary structure elements are listed in S1 Table.

The secondary structure of the 14-3-3ζ dimer remained mostly unchanged during our DF/HRE-MD and classical MD simulations, except for the occasional shortening of helix nine, and smaller conformational changes within the loops and disordered tails (e.g shown in S10 Fig). Secondary structure analyses of phosphopeptides for individual replicas of the DF/HRE-MD along the binding pathway were also performed using the DISICL algorithm. The secondary structure (SS) of phosphopeptides along their binding pathway was summarized for all eight DF/HRE-MD simulations in S11 and S12 Figs. In Fig 8A we present the DISICL profile of the p1h phosphopeptide along the binding pathway during the simulation dim_p1Ht. The phosphopeptide in IS1 (replicas 1–4) adopts an extended conformation, dominated by the β-cap (BC, brown) and polyproline-like (PP, maroon) classes (~60% of all amino acids in the ensemble). After the phosphopeptide leaves IS1 it adopts less extended conformations, the PP and BC classes become less characteristic, and the population of the helix-cap (HC, blue) class rises slightly.

The analysis of the other phosphopeptides revealed a similar highly extended backbone structure for all four phosphopeptide fragments in the bound state, with a dominance of the polyproline-like (PP) and β-cap (BC) classes. This is also in agreement with the conformations observed in the crystal structures and unrestrained MD simulations (a representative conformation is shown in Fig 8B). Similar trends were also observed for later stages of the eight DF/HRE-MD simulations. The presence of helical classes, mainly the helix-cap, π-helical (PIH, cyan), and α-helical (ALH, green) population was gradually increased to 5–20% as the peptide reached the unbound state, whilst the population of the extended classes (PC and BC) decreased to ~30%). The population of the secondary structure classes in the 100 ns MD simulations of the free phosphopeptide fragments (e.g shown in Fig 8C), also showed an increased helical preference (~20%) compared to the extended 14-3-3ζ-bound peptide fragments. In these simulations, the average population of π-helix is ~10%, significantly higher than what was expected for a random coil peptide (~0.4%). We found that this conformation was stabilized by intra-molecular interactions with the pSer sidechain.

Despite the common trends, phosphopeptide-specific conformational changes were also observed during the DF/HREMD simulations, with the largest conformational changes in secondary structure occurring between replicas 5–12 (Pw1, DF: 0.9–2.2 nm) and 20–25 (IS2, DF: 4.4–5.5 nm) along the binding pathway. The simulation dim_p2Ht showed an unusually high π-helical content, similar to the free peptide simulations. Note that dim_p2Ht did not follow the dominant binding pathway. The distorted π-helical conformation of the backbone in this simulation increased (to ~10%) gradually while the phosphopeptide gets unbound, stabilised by an intra-molecular hydrogen-bond bridge between the pSer 5 side-chain and residues His 2 and Arg 3. These interactions appear early in the pathway, when the phosphopeptide is prevented to follow the positive surface patch of the 14-3-3 dimer, due to the intervention of the C-terminal tail.

### 3. Free-energy changes along the binding pathway

#### 3.1. PMF profiles and binding affinity determination

**Fig 9:**
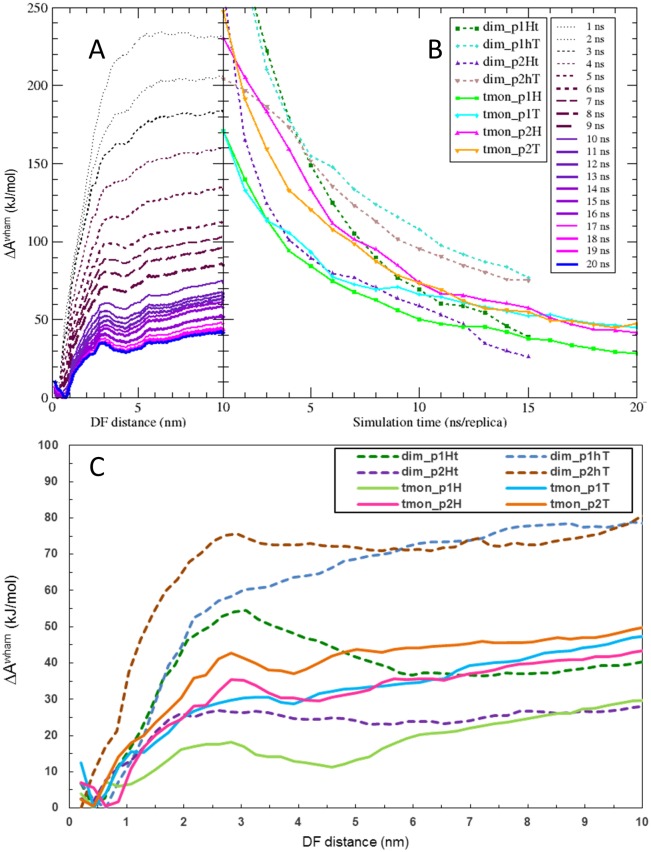
Free energy profiles of the DF/HRE-MD simulations. A) The raw free-energy profiles, derived from WHAM analysis, as function of simulation time per replica (every ns) for the simulation tmon_p2H. B) Convergence of the free energy difference between the unbound and bound state of all eight DF/HRE-MD simulations. C) Final free energy profiles (*ΔA*^*wham*^) for the eight 14-3-3ζ peptide-binding simulations.

The HRE-MD simulations were used to determine the Helmholtz free energy profiles (*A*^*wham*^(*l*)) along the binding pathway of the four phosphopeptide fragments bound to both dim and tmon models of 14-3-3ζ. It is important to emphasise that the DF/HRE-MD methodology greatly enhances the sampling of phosphopeptide binding/unbinding but it does not enhance the sampling of slow conformational transitions of the protein (e.g. C-terminal tail) to the same extent. Due to the slow transitions between different conformations, the simulations of dimeric complexes are not fully converged and should be considered only in a qualitative way (e.g. shape of curves *A*^*wham*^(*l*) in Fig 9). On the other hand, our minimalistic systems (tmon complexes) lack the C-terminal tail, sample a less complex conformational space and, therefore, the convergence of free energy profiles is much better. Fig 9A depicts the change of the free-energy profile (*A*^*wham*^(*l*)) over simulation time, as obtained from the weighted histogram analysis method (WHAM) for simulation tmon_p2h. The physical meaning of the free energy profile (*A*^*wham*^(*l*)) is that the probability (*ρ*(*l*)) of finding the phosphopeptide at the DF distance *l* is proportional to exp(-*A*^*wham*^(*l*)). The *A*^*wham*^(*l*) profiles for all systems were shifted by a constant such, that their minimal value is zero. Fig 9B presents the convergence of *A*^*wham*^(*l*) profiles as a function of DF/HRE-MD simulation time for the 8 studied complexes.

In order to calculate binding free energies that can be compared with the experimental binding affinities, a standard state correction has to be added to the raw WHAM profile as described in the Methods section (Eq 5). This correction relates the volume associated with the unbound state to the standard state volume. Table 1 shows the sampled and accessible volume (based on the restraining distance), as well as the approximate free energy from the WHAM calculations for every replica of simulation tmon_p2h, together with the zero-energy DF distance (*l*_*0*_). Once the unbound replicas are identified, the unbound volume is calculated from the sampled volume of the unbound replicas. The free energy of binding is calculated by subtracting the free energy of the unbound state (Eq 4) from the free energy of the bound state (Eq 3) and adding the standard state correction in accordance with Eq 5.

**Table 1:** Details of the DF/HRE-MD simulation of tmon_p2H. The table contains quantities for every replica that are required for the free energy calculations. The columns show the state assignment, the replica number, the sampled volume (*V*_*sampled*_), replica exchange probability (*P*_*ex*_), reaction coordinate value (*λ*), ideal distancefield distance (*l*_*0*_), accessible volume (*V(l)*) and raw free-energy according to the free-energy profile (*A*^*wham*^). *V(l)* and *A*^*wham*^ were calculated based on the restraining distance assigned to the replica. The last row contains the unbound volume (V_unb_), calculated from the volume sampled by the phosphopeptide in all Unb state replicas, and the volume of simulation box (V_box_).

State	Replica	*V*_*sampled*_	*P*_*ex*_	*λ*	*l*_*0*_	*V*(*l*)	*A*^*wham*^
tmon_p2H	Number	nm^3^			Nm	nm^3^	kJ/mol
IS1 (bound)	1	0.15	0.105	0.00	0.2	0.03	7
IS1 (bound)	2	0.27	0.223	0.02	0.4	0.09	5.6
IS1 (bound)	3	0.37	0.219	0.04	0.6	0.21	0.6
IS1 (bound)	4	0.44	0.222	0.06	0.9	0.60	1.7
IS1 (bound)	5	0.55	0.175	0.08	1.1	0.86	10.9
IS1/Pw1	6	0.75	0.099	0.11	1.3	0.77	16.7
IS1/Pw1	7	1.02	0.100	0.13	1.5	0.99	20.2
IS1/Pw1	8	1.38	0.130	0.15	1.7	1.21	22.9
Pw1	9	1.59	0.143	0.17	1.9	1.89	24.5
Pw1	10	1.60	0.123	0.19	2.2	2.19	28.1
Pw1	11	1.50	0.123	0.21	2.4	1.70	28.3
Pw1	12	1.78	0.118	0.23	2.6	2.02	32.2
IS2	13	2.05	0.089	0.25	2.8	2.47	35.4
IS2	14	2.65	0.102	0.27	3.0	2.99	35.2
IS2	15	2.71	0.121	0.29	3.3	5.61	33.9
IS2	16	2.66	0.130	0.32	3.5	7.00	31.8
IS2	17	2.78	0.113	0.34	3.7	5.84	30.3
IS2	18	2.72	0.098	0.36	3.9	6.93	30.3
IS2	19	3.19	0.137	0.38	4.1	10.94	29.8
IS2	20	2.78	0.170	0.40	4.4	13.14	29.5
IS2	21	3.35	0.156	0.42	4.6	10.47	30.5
IS2	22	4.12	0.120	0.44	4.8	12.05	31.1
IS2	23	4.91	0.119	0.46	5.0	13.82	31.7
IS2	24	5.61	0.150	0.48	5.2	15.69	32.6
IS2	25	6.76	0.128	0.51	5.5	27.42	34.7
IS2	26	7.37	0.114	0.53	5.7	31.08	35.7
IS2	27	7.69	0.142	0.55	5.9	22.56	35.4
Pw2	28	8.16	0.140	0.57	6.1	23.65	35.7
Pw2	29	7.67	0.132	0.59	6.3	24.40	35.7
Pw2/Unb	30	7.95	0.124	0.61	6.5	24.72	35.2
Pw2/Unb	31	9.47	0.125	0.63	6.8	24.76	36.2
Pw2/Unb	32	9.57	0.125	0.65	7.0	24.51	37
Pw2/Unb	33	9.75	0.111	0.67	7.2	23.97	37.5
Pw2/Unb	34	10.37	0.129	0.69	7.4	23.25	38.1
Unb (unbound)	35	10.69	0.138	0.72	7.6	22.52	39.1
Unb (unbound)	36	10.30	0.117	0.74	7.9	32.17	39.6
Unb (unbound)	37	9.83	0.114	0.76	8.1	30.47	39.9
Unb (unbound)	38	9.59	0.109	0.78	8.3	18.96	40.4
Unb (unbound)	39	10.17	0.112	0.80	8.5	17.80	40.7
Unb (unbound)	40	9.34	0.111	0.82	8.7	16.40	41
Unb (unbound)	41	8.84	0.107	0.84	9.0	15.00	40.9
Unb (unbound)	42	9.89	0.123	0.86	9.2	13.74	41.7
Unb (unbound)	43	9.90	0.124	0.88	9.4	12.47	41.8
Unb (unbound)	44	9.04	0.109	0.91	9.6	11.20	42.1
Unb (unbound)	45	7.60	0.064	0.93	9.8	9.98	42.9
Unb (unbound)	46	6.96	0.090	0.96	10.2	8.82	43.7
Unb (unbound)	47	7.73	0.134	0.98	10.4	7.74	43.9
Unb (unbound)	48	6.87	0.059	1.00	10.6	6.73	45
	V_unb_	74.8	nm^3^		V_box_	626.4	nm^3^

Table 2 summarises the resulting binding free energies and draws a comparison with the available experimental binding affinities. The binding affinities of 14-3-3ζ to very similar peptide fragments with identical binding recognition sequences were recently documented using fluorescence spectroscopy and isothermal calorimetry (ITC) methods [8,15], resulting in binding affinities in the 100–10 μM range corresponding to binding free energies of -20 to -30 kJ/mol. Surface plasmon resonance (SPR) experiments [2,10] on identical or very similar peptides reported binding affinities of about 100 nM, corresponding to binding free energies of -45 kJ/mol (p2t peptide). Our computational absolute binding affinities in the range of -30 to -55 kJ/mol correspond roughly to the available experimental data taking into consideration the fact that no fitting parameters were used. Please note that the presented experimental binding free-energies were measured for longer peptides with an identical recognition sequence, thus the free energies of binding do not need to agree completely with the ones calculated from our simulations. The monomeric PMF profiles as well as two of the dimeric profiles (dim_p1Ht and dim_p2hT) have a second, local free-energy minimum at 3.5–5.0 nm in the distancefield, associated with IS2 and a free energy barrier at ~3.0 nm separating IS1 and IS2 (Fig 9C). For the other two complexes (dim_p1hT and dim_p2Ht) neither the barrier nor the local minimum was observed, this is likely due to interference from the C-terminal tail of 14-3-3ζ, which was—in both cases—mostly located on the inner side of the 14-3-3 dimer.

**Table 2:** Comparison of the experimental and calculated binding free energies. The Δ*G*_*exp*_ shows the experimental free energies calculated from dissociation constants [8–10]. ΔA_bind_(mon) shows the binding free energy calculated from the tmon DF/HRE-MD simulations (20ns/replica).

Simulated	ΔG_exp_	ΔA_bind_(mon)
System	kJ/mol	kJ/mol
tmon_p1H	-28.8	-30.9
tmon_p1T	-23 *	-49.1
tmon_p2H	-21.6	-47.8
tmon_p2T	-27.4 / -44.2	-52.9

*: weak binding, estimate based on detection limit

#### 3.2. Impact of C-terminal tail on binding to the IS2

Comparison of free energy profiles between full length dimeric and truncated monomeric models of the 14-3-3ζ complexes allowed us to estimate the impact of the C-terminal tail on the phosphopeptide binding. The conformational transition of the C-terminal tail between the inner and outer side of 14-3-3ζ (Fig 2, S2 and S3 Tables) was a slow collective motion, poorly sampled in the dimeric simulations. However, two of DF/HRE-MD simulations (dim_p1Ht and dim_p2hT) thoroughly sampled the most probable binding pathways whilst the C-terminal tail remained attached to the outer protein surface. The other two simulations (dim_p1hT and dim_p2Ht) sampled pathways, during which the tail was attached to IS2 (more structural details in section 2.1).

The PMF profile of simulations where the C-terminal tail was for a considerable time present on the inner side of the 14-3-3ζ dimer (dim_p1hT and dim_p2Ht) does not exhibit a local free-energy minimum associated with IS2 (Fig 9C), or a free-energy barrier which would prevent the phosphopeptide to directly bind to IS1. In case of dim_p2Ht, the C-terminal tail was directly interacting with IS2 and prevented interactions between the p2h peptide and IS2. In case of dim_p1hT, IS2 was partially available for the p1t peptide, however, interactions between p1t and the C-terminal tail disrupted interactions with IS2. On the other hand, in simulations where the C-terminal tail was not present (all tmon simulations) or mostly present on the outer surfaces of 14-3-3ζ (dim_p1Ht and dim_p2hT) the drop in free-energy profile in the IS2 area and a free energy barrier (at ~3.0 nm separating IS1 and IS2) are present. These two features suggest an intermediate state (when the peptide is bound IS2) along the phosphopeptide binding pathway, which is diminished by the presence of the C-terminal tail near the inner side of the 14-3-3ζ dimer. This led us to the conclusion that one of the roles of the C-terminal tail may be to weaken the interaction between phosphopeptides and IS2 of the 14-3-3ζ protein.

## Conclusions

We explored the phosphopeptide binding pathways of the 14-3-3ζ protein through molecular dynamics simulations of four phosphopeptide fragments derived from PKC-ε and C-RAF kinase. The pathways were explored by a novel Hamiltonian replica exchange molecular dynamics method with incorporated distancefield restraints (DF/HRE-MD). The eight DF/HRE-MD simulations (4 dimeric and 4 truncated monomeric complexes) combined corresponded to more than 6.7 μs of enhanced-sampling simulation time, allowing for the unbiased determination of the most probable binding/unbinding pathways, the corresponding structural changes of the phosphopeptides and 14-3-3ζ, as well as the PMF profiles along the binding pathway.

The determined binding pathways were very similar for 7 out of the 8 studied complexes, suggesting a dominant phosphopeptide pathway, which roughly followed helix 3 between the primary binding site (IS1) and a newly identified secondary interaction site (IS2), localized in the second half of helix 3 (residues 60–70). We found that the flexible C-terminal tail of 14-3-3ζ may interact with both IS2 and the phosphopeptide in our simulations. When the C-terminal region interfered with the phosphopeptide binding pathway the interactions between the phosphopeptide and 14-3-3ζ IS2 were changed significantly, and this change was also reflected in the corresponding PMF profiles.

We confirmed previous findings suggesting that 14-3-3 monomers in complex with a phosphopeptide are shifted towards a more closed conformation as compared to the apo state. Our DF/HRE-MD simulations revealed that the 14-3-3ζ monomer adopts a wide-opened conformation when a phosphopeptide is to enter or leave IS1. The phosphopeptide secondary structure during the DF/HRE-MD simulations also changed from an extended conformation at IS1 into a less ordered structure with ~20% helical content, with a high probability of π-helical conformations in the unbound state. Sequence specific rearrangements in the peptide structure were detected during the Pw1 and IS2 stages, followed by the gradual increase of helical content as the phosphopeptides detached from 14-3-3ζ.

The DF/HRE-MD simulations allowed effective pathway sampling of the phosphopeptide fragments within the 14-3-3ζ protein. Application of distancefield restraints prevented the 14-3-3ζ protein damage that was observed in cases when regular distance-restraints were applied. While the full length 14-3-3ζ WT dimer models complexed with the four phosphopeptides proved to be useful for exploring the structural properties, their size and slow convergence prevented effective free-energy calculation for these systems. Therefore minimalistic models based on a truncated 14-3-3ζ monomer were designed, which proved to be more efficient in calculating the potential-of-mean-force (PMF) profiles for the binding of phosphopeptide/14-3-3ζ complexes. The binding free energies derived from the calculated PMF profiles of minimalistic 14-3-3ζ models show a reasonable agreement with known experimental binding affinities between similar PKC-ε and C-RAF kinase fragments and the 14-3-3ζ protein.

Taken together, these findings deepen our understanding about the binding phenomena of phosphopeptides to 14-3-3ζ and the obtained results likely have a wider applicability for other human isoforms considering their high sequence identity.

## Methods

All molecular dynamics (MD) and Hamiltonian replica exchange MD (HRE-MD) simulations were performed using the GROMOS11 software package [16]. The structure preparation and analysis of the simulation trajectories was based on the GROMOS++ analysis package [17]. The PyMol molecular graphics system version 1.5.0.3 [18] was used for visualization and calculation of the electrostatic surface potentials based on an adaptive Poisson-Boltzmann solver [19] as implemented in the PyMol software. Changes in the protein and peptide secondary structure were followed by the DISICL algorithm [20].

### Generated starting coordinates

The starting coordinates of the presented 14-3-3ζ models were generated based on the same crystal structure (pdb code: 2WH0) in order to prevent structural changes due to different protein starting structures. The crystal structure 2WH0 represents the dimeric 14-3-3ζ in complex with protein kinase C ε peptide fragments [8] (p1h, p1t). The structure of the C-RAF proto oncogene peptide fragments [9] (p2h, p2t) bound to 14-3-3ζ was taken from the structure of their co-crystal (pdb code: 4FJ3), after aligning the 14-3-3ζ dimer structures with 2WH0. The missing parts of the 14-3-3ζ protein, including the C-terminal tail, were added using Modeller version 9v8 [21]. The phosphopeptide structure was energy minimized in vacuum to avoid clashes with the 2WH0 protein atoms. The 14-3-3ζ dimer model without ligands was constructed based on the 2WH0 crystal structure where all peptide atoms were removed. The sequence of the complete peptides, as well as the head and tail fragments are indicated in Fig 1. The head and tail peptide fragments were chosen on the basis that they contain the consensus binding motif and the phosphorylation site in the middle of the sequence, consist of 8 amino acids for all peptide fragments, and have a total charge of -1.

Minimalistic (truncated monomeric) models were constructed from the dimer complexes by extracting the corresponding 14-3-3ζ monomer (along with its phosphopeptide) and truncating the last 15 amino acids (C-terminal stretch).

The prepared PDB coordinate files were transformed into GROMOS configuration files compatible with the GROMOS 54a7 force field [22], with phosphorylation parameters taken from the Vienna PTM 54a7 extension [23]. The structures were then energy minimised, solvated in pre-equilibrated SPC water [24] to fill a rectangular box with a minimal solute-to-wall distance of 2.0, 2.0, and 2.5 nm in the X, Y and Z dimensions, respectively, to provide sufficient space to pull the phosphopeptide fragments out of the binding site. The solute models were rotated in such a way that the largest dimension of the solute complex was oriented along the Z axis. Sodium and chloride ions were added to all simulation boxes to neutralise the total charge and provide a NaCl concentration of 0.15 or 0.25 M for electrostatic screening. The systems of the monomers typically contained 57 000 atoms, whilst the dimer complexes amounted to roughly 121 000 atoms. After solvation, the simulation boxes were energy minimised, then heated up from 60 to 298 K while gradually reducing the position restraints on protein and peptide atoms (initial force constant of 2.5 x 10^4^ kJ/mol/nm^2^) in 5 discrete steps of 20 ps each, and subsequently equilibrated for 60 ps without position restraints.

### Molecular dynamics simulations

Equilibration and all simulations were performed under periodic boundary conditions, using a 2-fs time step and the SHAKE algorithm [25] to constrain bond lengths and H–O–H bond angles. The weak-coupling algorithm [26] was used to maintain a stable temperature (300 K) and pressure (101 kPa) when required, with relaxation times of 0.1 and 0.5 ps respectively. For long-range interactions the reaction field method [27] was used with a 1.4 nm cut-off and 61 as dielectric permittivity [28]. During all of the production simulations roto-translational constraints [29] were kept on all protein atoms to prevent tumbling of the complexes in the rectangular simulation box.

During our simulations SHAKE errors occurred frequently at planar amide groups found in peptide bonds of the polypeptide chain and in the side chain amide groups with a delocalised character, such as arginines, glutamines and asparagines. The backbone N-H bond of arginine 18 appeared particularly regularly due to local structural strain. To treat this frequently occurring numerical problem, we increased the mass of this particular hydrogen by a factor of 5, in both our MD and HRE-MD production runs. MD simulations of all 14-3-3ζ dimer, monomer and complex systems were performed for 40 ns, and all phosphopeptide related MD simulations for 100 ns.

Preliminary simulations proved to be quite sensitive to the NaCl concentration. Our initial intention was to run all simulations at 0.15 M NaCl (physiological) concentration. This corresponds to the salt concentration at which most of experimental binding affinities were measured. However, preliminary simulations of the dimeric systems at 0.15 M showed significant instabilities in terms of inter-monomer twist angles, defined as the dihedral angle of the Cα atoms of the residues L43(M1)-A54(M1)-A54(M2)-L43(M2) where M1 and M2 indicate different monomers within the 14-3-3ζ dimer (Fig 7A). These problems were not observed at higher, 0.25 M NaCl concentration (S9A Fig). The comparison of experimental and calculated SAXS profiles of dim systems (S14 Fig) indicated that low values of inter-monomer twist (often observed at 0.15 M NaCl) do not agree with solution SAXS data. Therefore, we decided to perform all dimeric (dim) simulations (presented in this study) at 0.25 M NaCl and all tmon simulations at 0.15 M NaCl where inter-monomer twist instability cannot occur.

### Distancefield replica-exchange simulations

DF distance restraints and HRE-MD were applied as they are implemented in the GROMOS11 version 1.3.0 [16]. To compute the binding free energies for the phosphopeptide fragments (p1h, p1t, p2h, p2t), a reaction coordinate was defined as the DF distance between a virtual atom at the binding site of the corresponding 14-3-3ζ monomer and a virtual atom at the phosphorylation site of the peptide. The virtual atoms for all peptides were defined by the centre of mass of the C_β_ carbon atoms of the phosphorylated serine and its two neighbouring amino acids. The virtual atom for the binding site was defined as the centre of mass of the C_α_ atoms in residues N50, A192 and the amide hydrogen of N224 of the appropriate monomer. Simulations were started along the reaction coordinates to pull one of the peptides out of its respective binding pocket. This was done using a harmonic distancefield [6] distance restraint in 20 discrete steps with a minimal-energy distance ranging from 0.2 to 10.6 nm, a force constant of 2500 kJ/mol/nm^2^ and a simulation length of 250 ps at each step. The final configurations at each step were used to start 100 ps of HRE-MD [30] equilibration. During the HRE-MD equilibration with 48 replicas (Table 1) exchange events were prohibited and position restraints (25 kJ/mol/nm^2^) were applied to the protein atoms. After this equilibration, the final coordinates were then used to start the DF/HRE-MD production simulations, where replica exchanges were permitted every 10 ps and position restraints on the proteins were replaced with roto-translational constraints to maintain the protein orientation. Each of the four HRE-MD simulations of dim complexes was run for 15 ns; and of tmon complexes for 20 ns, using 48 replicas to cover a DF distance of ~10 nm from the IS1. The average interaction energy, and hydrogen bonds were monitored between peptide and protein atoms at each replica to determine the minimal distance for the unbound state (Fig 2C).

HRE-MD production simulations for each peptide fragment uniformly used a harmonic DF restraining potential with a force constant of 350 kJ/mol/nm^2^. The harmonic potential was linearised after deviations larger than 2 nm in order to avoid large forces over longer distances. The DF for the distance calculations was updated each 100 steps with a grid spacing and protein cut-off of 0.2 nm, and a single smoothing step to decrease repulsion at the protein surface [6]. HRE-MD simulations were kept at a constant temperature of 300 K by the weak coupling algorithm and were performed at a constant volume. The combined simulation time length of the DF/HRE-MD comes to a total of ~6.7 μs of enhanced sampling to study the 14-3-3ζ peptide binding.

### Phosphopeptide pathway assignment

The phosphopeptide binding-pathways obtained from DF/HRE-MD simulations were divided into 5 parts (IS1, Pw1, IS2, Pw2, Unb) during the analysis. The replicas of the DF/HRE-MD simulations were assigned to IS1 or IS2 based on three factors:

1. the average protein-phosphopeptide interaction energy of the replica had to be near a local minimum.
2. interactions observed with 14-3-3ζ residues in the corresponding interaction sites (more details below).
3. the most probable locations to find the phosphopeptide for the replica had to be in the proximity of the interaction site.

An interaction partner was considered stable in the interaction analysis if on average at least 0.1 interaction was present in the particular replica. Interaction partners for IS1 were K49, R127, Y128 towards the pS of the peptide. Interaction partners for IS2 were K68 towards the pS and any interaction involving S64-G70. Proximity from an interaction site was determined by measuring the distance between density peak maxima of the phospopeptide virtual atom for that particular replica (as shown in Fig 8) and Cα of K49 and K68 for IS1 and IS2, respectively. The density peak was considered proximal within 1.5 nm. The replicas were assigned to unbound state (Unb) if the interaction energy was close to zero and no stable hydrogen-bonds were detected between the phosphopeptide and 14-3-3ζ. Replicas that were fulfilling only part of the criteria were assigned to Pw1 and Pw2 instead. An example of assignment is show in Table 1, and replica density peaks are coloured according to their assignment in Fig 2, S1–S7 Figs.

### Free energy calculations

The free energy profile was calculated from the replica exchange simulations, using a weighted histogram analysis method (WHAM) [31]. The WHAM is an iterative method that uses the probability distribution of biased ensembles $\left(\rho_{i}^{\left(b\right)}\right)$ and reconstructs the unbiased probability distribution (*ρ*) by fitting the free-energy of a given number of windows (*Nw*) along the reaction coordinate. Using the DF distance distributions of the replicas—biased by the harmonic DF restraining potentials (*B*_*j*_)–the probability distribution along the peptide binding pathway is calculated iteratively according to Eqs 1 and 2:

$$\rho \left(l\right)=\frac{\sum_{i=1}^{Nw}n_{i}\;\rho_{i}^{\left(b\right)}\left(l\right)}{\sum_{j=1}^{Nw}n_{j}e^{-\left(B_{j}\left(l\right)-A_{j}^{wham}\right)/RT}}$$ (1)

$$e^{-A_{j}^{lwham}/RT}=\;\int \rho \left(l\right)\;e^{-B_{j}\left(l\right)/RT}dl$$ (2)

Where *ρ(l)* is the probability of finding the peptide at the DF distance *l*, $A_{j}^{wham}$ is the Helmholtz free energy at the DF window j, *n*_*i*_ is the number of data points in replica i, R is the ideal gas constant, and *T* is the temperature. The resulting free-energy profile(*A*^*wham*^(*l*)) was used to calculate the free energy of the bound (*A*_*bound*_) and unbound (*A*_*unb*_) states, by integrating the profile over the DF distances associated with the two states, as shown in Eqs 3 and 4, respectively:

$$A_{bound}=-RT\text{ln}\underset{}{\int_{bound}}e^{-A^{wham}\left(l\right)/RT}\;dl$$ (3)

$$A_{unb}=-RT\text{ln}\underset{}{\underset{}{\int_{unb}}}e^{-A^{wham}\left(l\right)/RT}\;dl$$ (4)

Because of the shape of *A*^*wham*^(*l*) in the bound area the *A*_*bound*_ value is quite insensitive on the particular choice of boundaries of the integral in Eq 3. On the other hand, *A*_*unb*_ depends significantly on the particular choice of the *unb* region boundaries in Eq 4. This dependence is mainly due to entropic contributions from the increased available volume of the unbound state, and is compensated by the standard state correction (*ΔA*_*std*_) in the final calculation of the binding free energy (*ΔA*_*bind*_) by Eq (5):

$$\Delta A_{bind}=A_{bound}-A_{unb}+\Delta A_{std};\Delta A_{std}=-RT\text{ln}\frac{V_{unb}}{V_{0}}$$ (5)

where *V*_*0*_ is the standard state volume (1.66 nm^3^) corresponding to a 1 M concentration. The physical meaning of Eq 5 is that the binding free energy (*ΔA*_*bind*_) corresponds to the free-energy difference of bringing a ligand from a bound to an unbound state and subsequently from the unbound to the standard state volume [32]. ΔA_bind_ can be directly compared with experimental values calculated from dissociation constants. The unbound volume (*V*_*unb*_) was calculated based on the number of grid-points visited by the phosphopeptide virtual atom in any replica of unbound state (Table 1).

To calculate the free-energy profiles of the peptide binding processes, we used the DF distributions of the DF distances collected over the 48 replicas of the HRE-MD simulations and 100 DF widows for the iterative WHAM process, which was continued for 10 000 steps or until the energy change was less than 10^−5^ kJ/mol. Calculations of the available volume were approximated from the DF grid based on the number of gridpoints assigned to a given distance. The volumes sampled for the replica exchange simulations were calculated based on a similar grid with a mesh size identical to DF grids (0.2 nm) where the movements of the peptide virtual atom were tracked. By recording which gridpoint was the closest to the peptide virtual atom position at each frame (after every 5 ps of simulation), we could determine the probability density of the given gridpoint (*P*_*i*_). To find the conformations most often visited by the peptide, the relative probability density $\left(P_{i}^{r}\right)$ of the gridpoint *i* was calculated according to Eq 6:

$$P_{i}^{r}=\frac{P_{i}-P_{min}}{P_{max}-P_{min}}\;\;\;P_{i}=\frac{O_{i}}{N}$$ (6)

**Fig 10:**
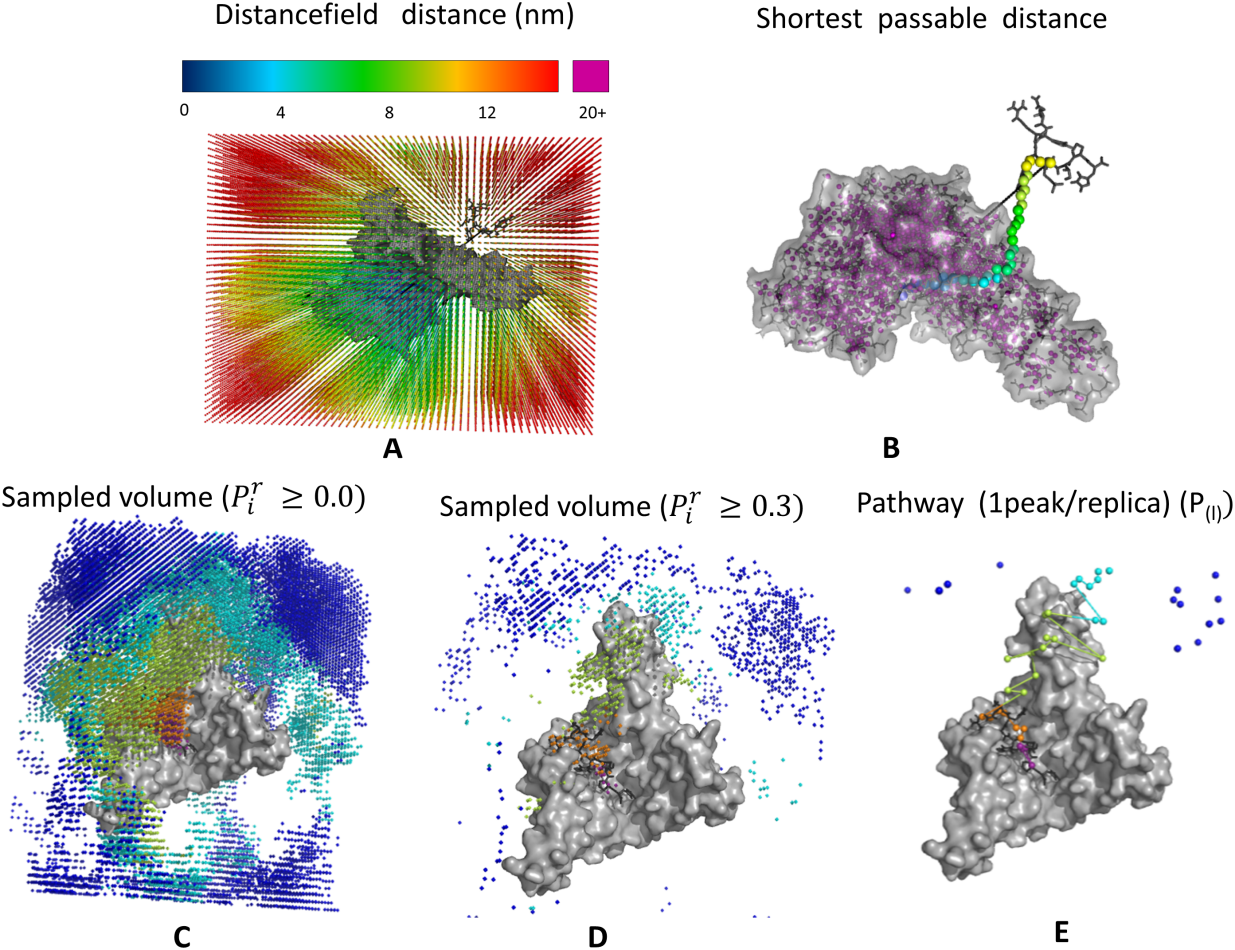
Graphical representation of pathway mapping in simulations. A) Distancefield grid, where grid points are coloured according to distancefield (DF) distance. B) Direct distance (as a black dashed line), and DF distance (along the coloured grid points) between the ligand and the protein binding side. Grid points located within the protein (which should be avoided) are shown in purple. C-E) Grid points sampled during a replica exchange simulation, coloured according to their position along the pathway. Panels C and D are showing grid points at different levels of relative probability $\left(P_{i}^{r}\right)$ per replica; with all visited grid points in Panel C, and only the often-visited grid points per replica in Panel D. Panel E shows the derived peptide-binding pathway, with one peak maximum per replica. The surface of the protein is shown in grey.

Where *P*_*max*_ and *P*_*min*_ are the probabilities of visiting the least and most often visited gridpoints, *O*_*i*_ is the number of times the gridpoint was visited and *N* is the number of simulation frames used in the calculation (an example is shown in Fig 10).

## Supporting information

S1 Table DISICL secondary structure elements. Structure elements and abbreviations are listed below. For a detailed description of the DISICL classes see [20].
(DOCX)

S2 Table C-terminal tail distributions in DF/HRE-MD simulations. The table displays the name of the simulation, the identity of the monomer (mon), the probability to find the C-terminal tail near the primary binding site (IS1), secondary binding site (IS2), on the outer protein surface (out), and free in solution (sol) over the entire length of the simulation, along the total number of transitions between the listed sub-states (observed transitions) Note that the phosphopeptide fragments p1h and p2t were binding to M1, whilst the p1t and p2h were binding to M2.
(DOCX)

S3 Table C-terminal tail distribution in unrestrained MD simulations. The table displays the name of the simulation, the identity of the monomer (mon), the probability to find the C-terminal tail near the primary binding site (IS1), secondary binding site (IS2), on the outer protein surface (out), and free in solution (sol) over the entire length of the simulation, along with the total number of transitions between the listed sub-states (Observed transitions) Note that simulations were started with the M1 tail close to the outer surface and the M2 tail close to the inner surface (near IS2). Dim-2 denotes dimeric 14-3-3ζ- simulation started from an alternative set of starting coordinates.
(DOCX)

S1 Fig Pathway visualization of the simulation tmon_p1H. A) The volume sampled by the p1h phosphopeptide during the simulation is shown by dots around the 14-3-3ζ protein coloured based on their position along the pathway. The most probable points in space to find the peptide in for each replica (probability density peaks) are represented by larger spheres. B) Density peaks and a few representative structures corresponding to the density peaks, coloured according to their position along the pathway. Replica density peaks are connected by lines to visualize the binding pathway. The 14-3-3ζ protein in panels A-B is shown as a surface representation, with the two monomers shown in light and dark grey. C) Average interaction energy between 14-3-3ζ and the p1h. D) Interaction map between any atom of the pulled p1h peptide and the amino acids of the protein, summarized for each replica (only amino acids with at least 0.1 hydrogen bond/salt bridge on average are shown). The scale indicates the number of interactions.
(TIF)

S2 Fig Pathway visualization of the simulation tmon_p2H. A) The volume sampled by the p2h phosphopeptide during the simulation is shown by dots around the 14-3-3ζ protein coloured based on their position along the pathway. The most probable points in space to find the peptide in for each replica (probability density peaks) are represented by larger spheres. B) Density peaks and a few representative structures corresponding to the density peaks, coloured according to their position along the pathway. Replica density peaks are connected by lines to visualize the binding pathway. The 14-3-3ζ protein in panels A-B is shown as a surface representation, with the two monomers shown in light and dark grey. C) Average interaction energy between 14-3-3ζ and the p2h. D) Interaction map between any atom of the pulled p2h peptide and the amino acids of the protein, summarized for each replica (only amino acids with at least 0.1 hydrogen bond/salt bridge on average are shown). The scale indicates the number of interactions.
(TIF)

S3 Fig Pathway visualization of the simulation tmon_p1T. A) The volume sampled by the p1t phosphopeptide during the simulation is shown by dots around the 14-3-3ζ protein coloured based on their position along the pathway. The most probable points in space to find the peptide in for each replica (probability density peaks) are represented by larger spheres. B) Density peaks and a few representative structures corresponding to the density peaks, coloured according to their position along the pathway. Replica density peaks are connected by lines to visualize the binding pathway. The 14-3-3ζ protein in panels A-B is shown as a surface representation, with the two monomers shown in light and dark grey. C) Average interaction energy between 14-3-3ζ and the p1t. D) Interaction map between any atom of the pulled p1t peptide and the amino acids of the protein, summarized for each replica (only amino acids with at least 0.1 hydrogen bond/salt bridge on average are shown). The scale indicates the number of interactions.
(TIF)

S4 Fig Pathway visualization of the simulation tmon_p2T. A) The volume sampled by the p2t phosphopeptide during the simulation is shown by dots around the 14-3-3ζ protein coloured based on their position along the pathway. The most probable points in space to find the peptide in for each replica (probability density peaks) are represented by larger spheres. B) Density peaks and a few representative structures corresponding to the density peaks, coloured according to their position along the pathway. Replica density peaks are connected by lines to visualize the binding pathway. The 14-3-3ζ protein in panels A-B is shown as a surface representation, with the two monomers shown in light and dark grey. C) Average interaction energy between 14-3-3ζ and the p2t. D) Interaction map between any atom of the pulled p2t peptide and the amino acids of the protein, summarized for each replica (only amino acids with at least 0.1 hydrogen bond/salt bridge on average are shown). The scale indicates the number of interactions.
(TIF)

S5 Fig Pathway visualization of the simulation dim_p1Ht. A) The volume sampled by the p1h phosphopeptide during the simulation is shown by dots around the 14-3-3ζ protein coloured based on their position along the pathway. The most probable points in space to find the peptide in for each replica (probability density peaks) are represented by larger spheres. B) Density peaks and a few representative structures corresponding to the density peaks, coloured according to their position along the pathway. Replica density peaks are connected by lines to visualize the binding pathway. The 14-3-3ζ protein in panels A-B is shown as a surface representation, with the two monomers shown in light and dark grey. C) Average interaction energy between 14-3-3ζ and the p1h. D) Interaction map between any atom of the pulled p1h peptide and the amino acids of the protein, summarized for each replica (only amino acids with at least 0.1 hydrogen bond/salt bridge on average are shown). The scale indicates the number of interactions.
(TIF)

S6 Fig Pathway visualization of the simulation dim_p2hT. A) The volume sampled by the p2t phosphopeptide during the simulation is shown by dots around the 14-3-3ζ protein coloured based on their position along the pathway. The most probable points in space to find the peptide in for each replica (probability density peaks) are represented by larger spheres. B) Density peaks and a few representative structures corresponding to the density peaks, coloured according to their position along the pathway. Replica density peaks are connected by lines to visualize the binding pathway. The 14-3-3ζ protein in panels A-B is shown as a surface representation, with the two monomers shown in light and dark grey. C) Average interaction energy between 14-3-3ζ and the p2t. D) Interaction map between any atom of the pulled p2t peptide and the amino acids of the protein, summarized for each replica (only amino acids with at least 0.1 hydrogen bond/salt bridge on average are shown). The scale indicates the number of interactions.
(TIF)

S7 Fig Pathway visualization of the simulation dim_p2Ht. A) The volume sampled by the p2h phosphopeptide during the simulation is shown by dots around the 14-3-3ζ protein coloured based on their position along the pathway. The most probable points in space to find the peptide in for each replica (probability density peaks) are represented by larger spheres. B) Density peaks and a few representative structures corresponding to the density peaks, coloured according to their position along the pathway. Replica density peaks are connected by lines to visualize the binding pathway. The 14-3-3ζ protein in panels A-B is shown as a surface representation, with the two monomers shown in light and dark grey. C) Average interaction energy between 14-3-3ζ and the p2h. D) Interaction map between any atom of the pulled p2h peptide and the amino acids of the protein, summarized for each replica (only amino acids with at least 0.1 hydrogen bond/salt bridge on average are shown). The scale indicates the number of interactions. Note that dim_p2Ht did not follow the general pathway observed for other DF/HRE-MD simulations, and IS2 residues were not significant interaction partners.
(TIF)

S8 Fig Interaction map between the perturbed phosphopeptides and amino acids of 14-3-3ζ. The map shows an average over the 8 HRE-MD simulations, where the average number of hydrogen bonds observed during the simulations are shown as the function of replica number. Replica 1–3 refers to the bound state in interaction site1 (IS1), and larger replica numbers correspond to higher DF distance from IS1.
(TIF)

S9 Fig The salt concentration dependence of the 14-3-3ζ inter-monomer twist. A) The drift of the monomer twist angle during MD simulations. B) Atom-positional root-mean-square deviation (RMSD) from the original conformation (derived from the crystal structure) during the various simulations. The structural deviation calculated for the individual monomers of simulation X are marked as X_M1 and X_M2, respectively.
(TIF)

S10 Fig 14-3-3ζ secondary structure analysis. DISICL backbone analysis shows a stable secondary structure during the MD simulation dim. The left side of the Figure shows a schematic representation of the 14-3-3ζ helices. The most populated DISICL classes are depicted in the following colours: α-helix (ALH)–green, π-helix (PIH)–cyan, helix-cap (HC)–blue, turn-cap (TC)–black, polyproline-like (PP)–brown, turn type I (TI)–magenta, Turn type II (TII)–purple. For a description of abbreviations of DISICL secondary structure elements see S1 Table.
(TIF)

S11 Fig Pathway dependence of phosphopeptide secondary structure. The population of secondary structure elements is dependent on the replica ID (and DF distance) within the dim DF/HRE-MD simulations. The change of the backbone secondary structure content was analysed by the DISICL algorithm. For a description of abbreviations of DISICL secondary structure elements see S1 Table.
(TIF)

S12 Fig Pathway dependence of phosphopeptide secondary structure. The population of secondary structure elements is dependent on the replica ID (and DF distance) within the tmon DF/HRE-MD simulations. The change of the backbone secondary structure content was analysed by the DISICL algorithm. For a description of abbreviations of DISICL secondary structure elements see S1 Table.
(TIF)

S13 Fig The effect of phosphorylation of Ser58 on the electrostatic surface potential (ESP) of 14-3-3ζ. The IS1 and IS2 in A) 14-3-3ζ WT are connected by a mildly positive surface potential patch in contrary to B) 14-3-3ζ phosphorylated at S58, where the connection is disrupted by the negatively charged phosphoserine. Blue, white and red colour represents the positive, neutral, and negative surface patches, respectively. For the clarity, the 14-3-3ζ C-terminal tails are not shown.
(TIF)

S14 Fig SAXS comparison. **Small angle** X-ray **scattering c**alculated from the crystal structure (cryst, pdb code 2WHO), the IS1 and UnB stages dim_p1hT, and the unrestrained MD simulations with a low intermonomer twist angles (dim_0.15 dim_p1ht_0.15). The_SAXS curves were calculated using the software Crysol (1), and compared to the experimental SAXS curve (exp) after ensemble averaging. The full length 14-3-3ζ protein sample used for SAXS measurements was expressed and purified as described in Hritz et al.[4]. SAXS data were collected on the beamline BM29 BioSAXS ESFR in Grenoble, France. The concentrations of the 14-3-3-WT during the measurement were 1.18; 2.35 and 4.70 mg/ml in the 50 mM Tris buffer, pH = 8.0. The data were recorded at 20.12°C using the pixel 1M PILATUS detector at a sample-detector distance of 2.867 m, and a wavelength (λ) of 0.099 nm, covering the range of momentum transfer 0.025 nm^−1^ < s < 5 nm^−1^ (s = 4π sin(θ)/λ, where 2θ is the scattering angle). No radiation damage was observed during the data collection.The data were processed using standard procedures with the PRIMUS software (2). Solvent contributions (buffer backgrounds collected before and after the protein sample) were averaged and subtracted from the associated protein sample. A slight concentration dependency was noticeable. Therefore, the scattering curves collected at different concentrations were used to obtain a final zero concentration scattering curve through extrapolation according to the guidelines provided by (3).
(TIF)
