# Supplementary material for: Exploring the binding pathways of the 14-3-3ζ protein: Structural and free-energy profiles revealed by Hamiltonian replica exchange molecular dynamics with distancefield distance restraints
Source: PLoS One. 2017 Jul 20;12(7):e0180633. doi: 10.1371/journal.pone.0180633 (PMC5519036; doi:10.1371/journal.pone.0180633)
Supplement: S1 Table — Structure elements and abbreviations are listed below. For a detailed description of the DISICL classes see [20]. (DOCX) [file pone.0180633.s001.docx]

| **Structure Element** | **Code** |
| --- | --- |
| 3/10-Helix | 3H |
| Turn type 1 | TI |
| Turn-Cap | TC |
| α-helix | ALH |
| π-Helix | PIH |
| Helix-Cap | HC |
| Ext. β-Strand | EBS |
| Normal β-Strand | NBS |
| β-Cap | BC |
| PP Helical | PP |
| Beta Bulge | BU |
| Turn type 2 | TII |
| Turn type 8 | TVIII |
| Gamma Turns | GXT |
| Schellman Turn | SCH |
| Hairpin 2:2 | HP |
| Left Turn 2 | LTII |
| Left-handed Helix | LHH |
