# Supplementary material for: Exploring the binding pathways of the 14-3-3ζ protein: Structural and free-energy profiles revealed by Hamiltonian replica exchange molecular dynamics with distancefield distance restraints
Source: PLoS One. 2017 Jul 20;12(7):e0180633. doi: 10.1371/journal.pone.0180633 (PMC5519036; doi:10.1371/journal.pone.0180633)
Supplement: S2 Table — The table displays the name of the simulation, the identity of the monomer (mon), the probability to find the C-terminal tail near the primary binding site (IS1), secondary binding site (IS2), on the outer protein surface (out), and free in solution (sol) over the entire length of the simulation, along the total number of transitions between the listed sub-states (observed transitions) Note that the phosphopeptide fragments p1h and p2t were binding to M1, whilst the p1t and p2h were binding to M2. (DOCX) [file pone.0180633.s002.docx]

| System | | Probability distribution | | | | Observed transitions | | | | | |
| --- | --- | --- | --- | --- | --- | --- | --- | --- | --- | --- | --- |
| REMD statistics | mon. | 1-IS1 (%) | 2-IS2 (%) | 3-out (%) | 4-sol (%) | 1-2 | 1-3 | 1-4 | 2-3 | 2-4 | 3-4 |
| dim_p1Ht Unb | M1 | 0.0 | 0.0 | 87.4 | 12.6 | 0 | 0 | 0 | 0 | 0 | 188 |
|  | M2 | 0.0 | 75.9 | 0.0 | 24.0 | 4 | 0 | 0 | 0 | 174 | 0 |
| dim_p1Ht IS1 | M1 | 0.0 | 0.0 | 73.0 | 27.0 | 0 | 0 | 0 | 0 | 0 | 145 |
|  | M2 | 0.0 | 76.4 | 12.8 | 10.9 | 0 | 0 | 0 | 96 | 80 | 16 |
| dim_p1hT Unb | M1 | 0.0 | 0.0 | 81.2 | 18.8 | 0 | 0 | 0 | 0 | 0 | 197 |
|  | M2 | 5.9 | 80.4 | 3.1 | 10.5 | 20 | 6 | 4 | 8 | 92 | 8 |
| dim_p1hT IS1 | M1 | 0.0 | 0.0 | 73.5 | 26.5 | 0 | 0 | 0 | 0 | 0 | 207 |
|  | M2 | 0.0 | 79.6 | 1.6 | 18.8 | 0 | 0 | 0 | 14 | 112 | 4 |
| dim_p2Ht Unb | M1 | 0.0 | 0.0 | 73.4 | 26.6 | 0 | 0 | 0 | 0 | 0 | 261 |
|  | M2 | 2.7 | 82.5 | 2.1 | 12.8 | 28 | 3 | 17 | 32 | 123 | 15 |
| dim_p2Ht IS1 | M1 | 0.0 | 0.0 | 90.2 | 9.8 | 0 | 0 | 0 | 0 | 0 | 46 |
|  | M2 | 0.0 | 84.2 | 4.4 | 11.4 | 0 | 0 | 0 | 28 | 52 | 4 |
| dim_p2hT Unb | M1 | 0.0 | 0.0 | 71.9 | 28.1 | 0 | 0 | 0 | 0 | 0 | 297 |
|  | M2 | 0.0 | 85.8 | 0.3 | 13.9 | 0 | 0 | 0 | 6 | 184 | 4 |
| dim_p2hT IS1 | M1 | 0.0 | 0.0 | 76.4 | 23.6 | 0 | 0 | 0 | 0 | 0 | 265 |
|  | M2 | 0.0 | 76.6 | 5.9 | 17.4 | 0 | 0 | 0 | 36 | 188 | 6 |
