# Supplementary material for: Exploring the binding pathways of the 14-3-3ζ protein: Structural and free-energy profiles revealed by Hamiltonian replica exchange molecular dynamics with distancefield distance restraints
Source: PLoS One. 2017 Jul 20;12(7):e0180633. doi: 10.1371/journal.pone.0180633 (PMC5519036; doi:10.1371/journal.pone.0180633)
Supplement: S3 Table — The table displays the name of the simulation, the identity of the monomer (mon), the probability to find the C-terminal tail near the primary binding site (IS1), secondary binding site (IS2), on the outer protein surface (out), and free in solution (sol) over the entire length of the simulation, along with the total number of transitions between the listed sub-states (Observed transitions) Note that simulations were started with the M1 tail close to the outer surface and the M2 tail close to the inner surface (near IS2). Dim-2 denotes dimeric 14-3-3ζ- simulation started from an alternative set of starting coordinates. (DOCX) [file pone.0180633.s003.docx]

| System | | Probability distribution | | | | Observed transitions | | | | | | | | | | | |
| --- | --- | --- | --- | --- | --- | --- | --- | --- | --- | --- | --- | --- | --- | --- | --- | --- | --- |
| MD statistics | mon. | 1-IS1 (%) | 2-IS2 (%) | 3-out (%) | 4-sol (%) | 1-2 | | 1-3 | | 1-4 | | 2-3 | | 2-4 | | 3-4 | |
| dim_p2ht | M1 | 0.0 | 0.0 | 69.8 | 30.2 | 0 | 0 | | 0 | | 0 | | 0 | | 72 | |  |
|  | M2 | 0.0 | 97.1 | 0.0 | 2.9 | 0 | 0 | | 0 | | 0 | | 16 | | 0 | |  |
| dim_p1ht | M1 | 0.0 | 0.0 | 77.9 | 22.1 | 0 | 0 | | 0 | | 0 | | 0 | | 87 | |  |
|  | M2 | 0.4 | 15.0 | 71.8 | 12.9 | 0 | 4 | | 2 | | 0 | | 15 | | 45 | |  |
| Dim | M1 | 0.0 | 0.0 | 73.8 | 26.2 | 0 | 0 | | 0 | | 0 | | 0 | | 38 | |  |
|  | M2 | 0.9 | 7.9 | 47.6 | 43.6 | 0 | 0 | | 4 | | 0 | | 17 | | 48 | |  |
| dim-2 | M1 | 0.0 | 0.0 | 73.0 | 27.0 | 0 | 0 | | 0 | | 0 | | 0 | | 101 | |  |
|  | M2 | 0.0 | 0.0 | 89.9 | 10.1 | 0 | 0 | | 0 | | 0 | | 0 | | 45 | |  |
