# Supplementary figures and images for: Exploring the binding pathways of the 14-3-3ζ protein: Structural and free-energy profiles revealed by Hamiltonian replica exchange molecular dynamics with distancefield distance restraints
Source: PLoS One. 2017 Jul 20;12(7):e0180633. doi: 10.1371/journal.pone.0180633 (PMC5519036; doi:10.1371/journal.pone.0180633)

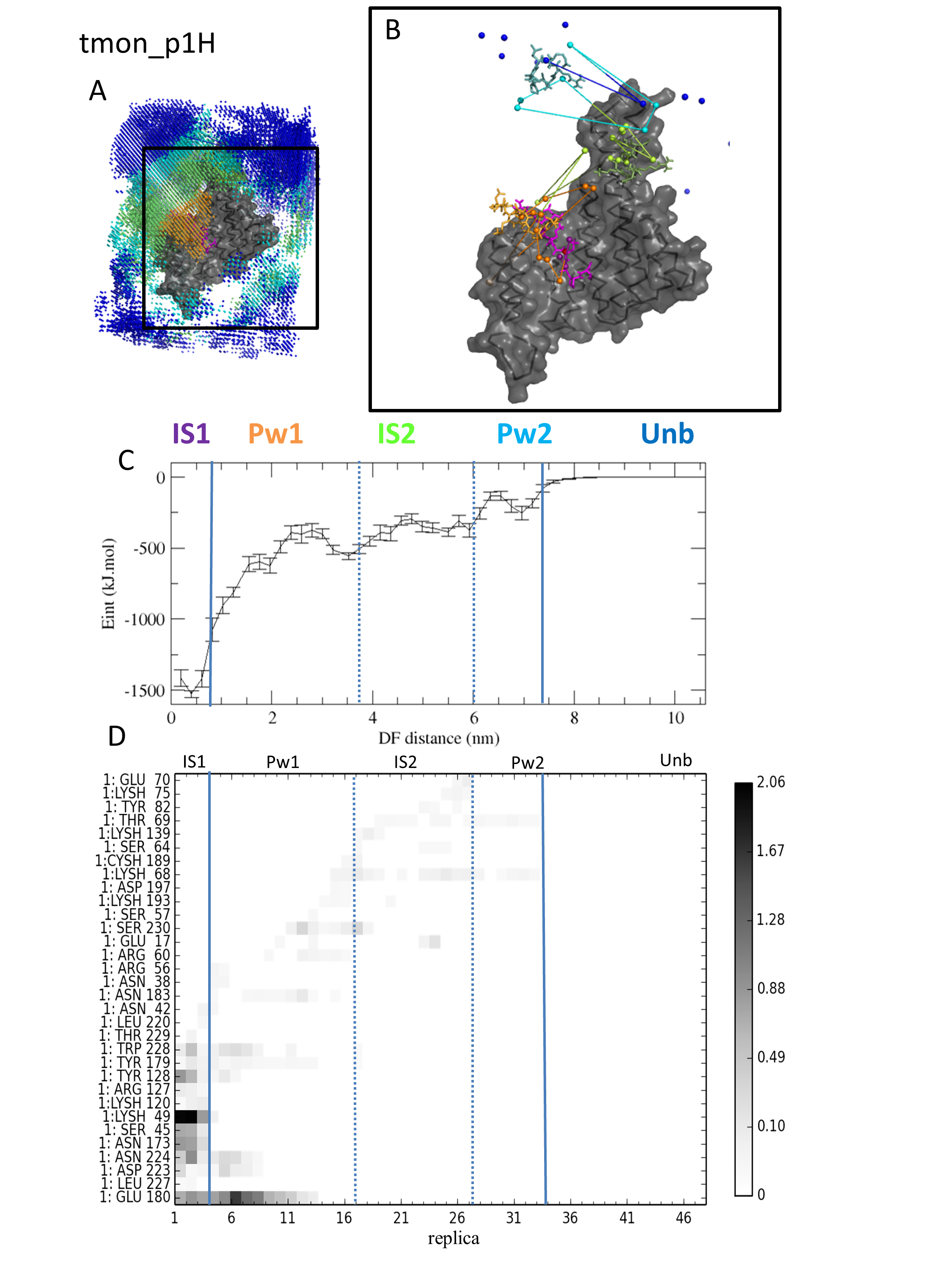

Supplement: S1 Fig — A) The volume sampled by the p1h phosphopeptide during the simulation is shown by dots around the 14-3-3ζ protein coloured based on their position along the pathway. The most probable points in space to find the peptide in for each replica (probability density peaks) are represented by larger spheres. B) Density peaks and a few representative structures corresponding to the density peaks, coloured according to their position along the pathway. Replica density peaks are connected by lines to visualize the binding pathway. The 14-3-3ζ protein in panels A-B is shown as a surface representation, with the two monomers shown in light and dark grey. C) Average interaction energy between 14-3-3ζ and the p1h. D) Interaction map between any atom of the pulled p1h peptide and the amino acids of the protein, summarized for each replica (only amino acids with at least 0.1 hydrogen bond/salt bridge on average are shown). The scale indicates the number of interactions. (TIF) [file pone.0180633.s004.TIF]

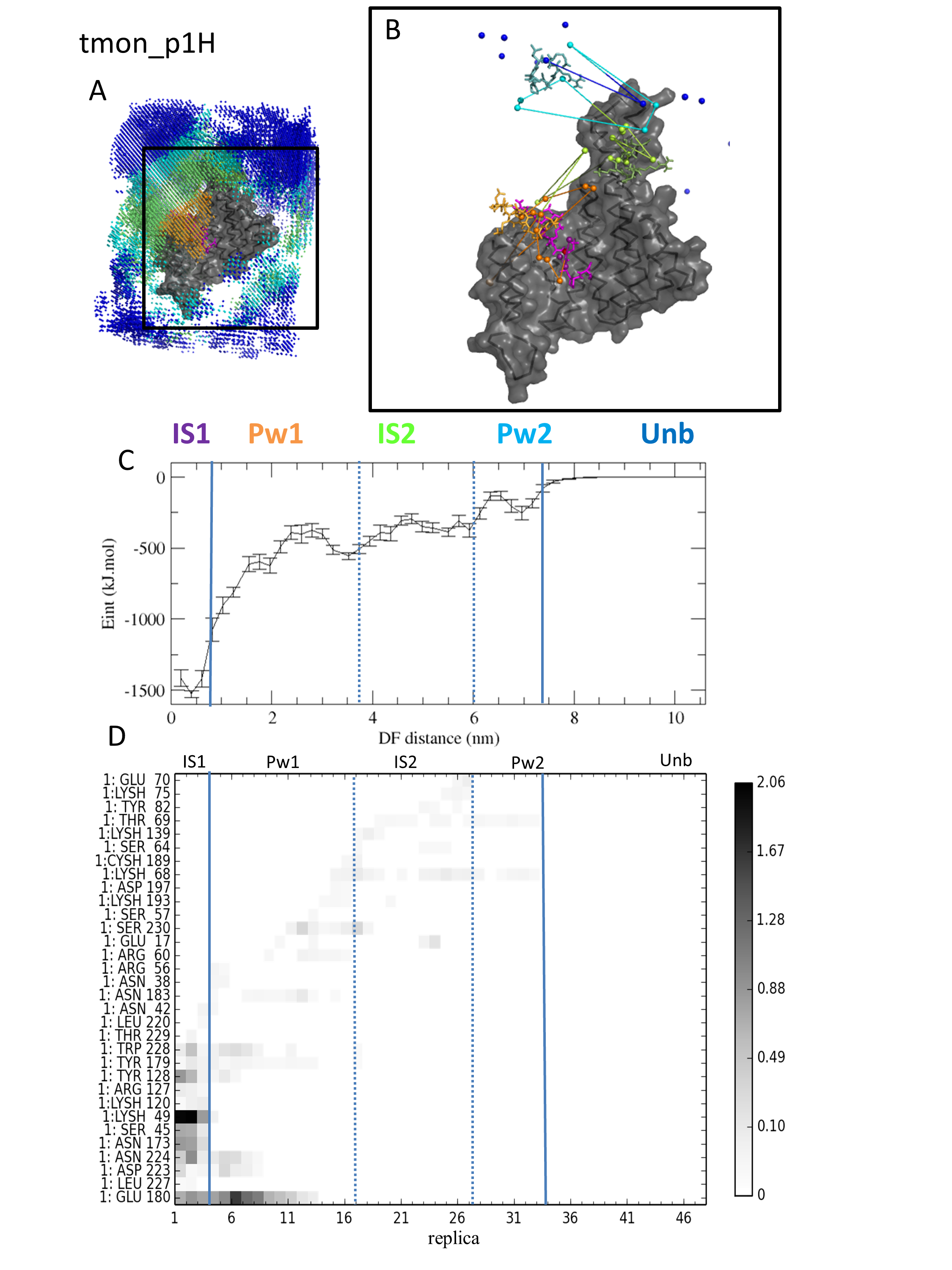

Supplement: S2 Fig — A) The volume sampled by the p2h phosphopeptide during the simulation is shown by dots around the 14-3-3ζ protein coloured based on their position along the pathway. The most probable points in space to find the peptide in for each replica (probability density peaks) are represented by larger spheres. B) Density peaks and a few representative structures corresponding to the density peaks, coloured according to their position along the pathway. Replica density peaks are connected by lines to visualize the binding pathway. The 14-3-3ζ protein in panels A-B is shown as a surface representation, with the two monomers shown in light and dark grey. C) Average interaction energy between 14-3-3ζ and the p2h. D) Interaction map between any atom of the pulled p2h peptide and the amino acids of the protein, summarized for each replica (only amino acids with at least 0.1 hydrogen bond/salt bridge on average are shown). The scale indicates the number of interactions. (TIF) [file pone.0180633.s005.TIF]

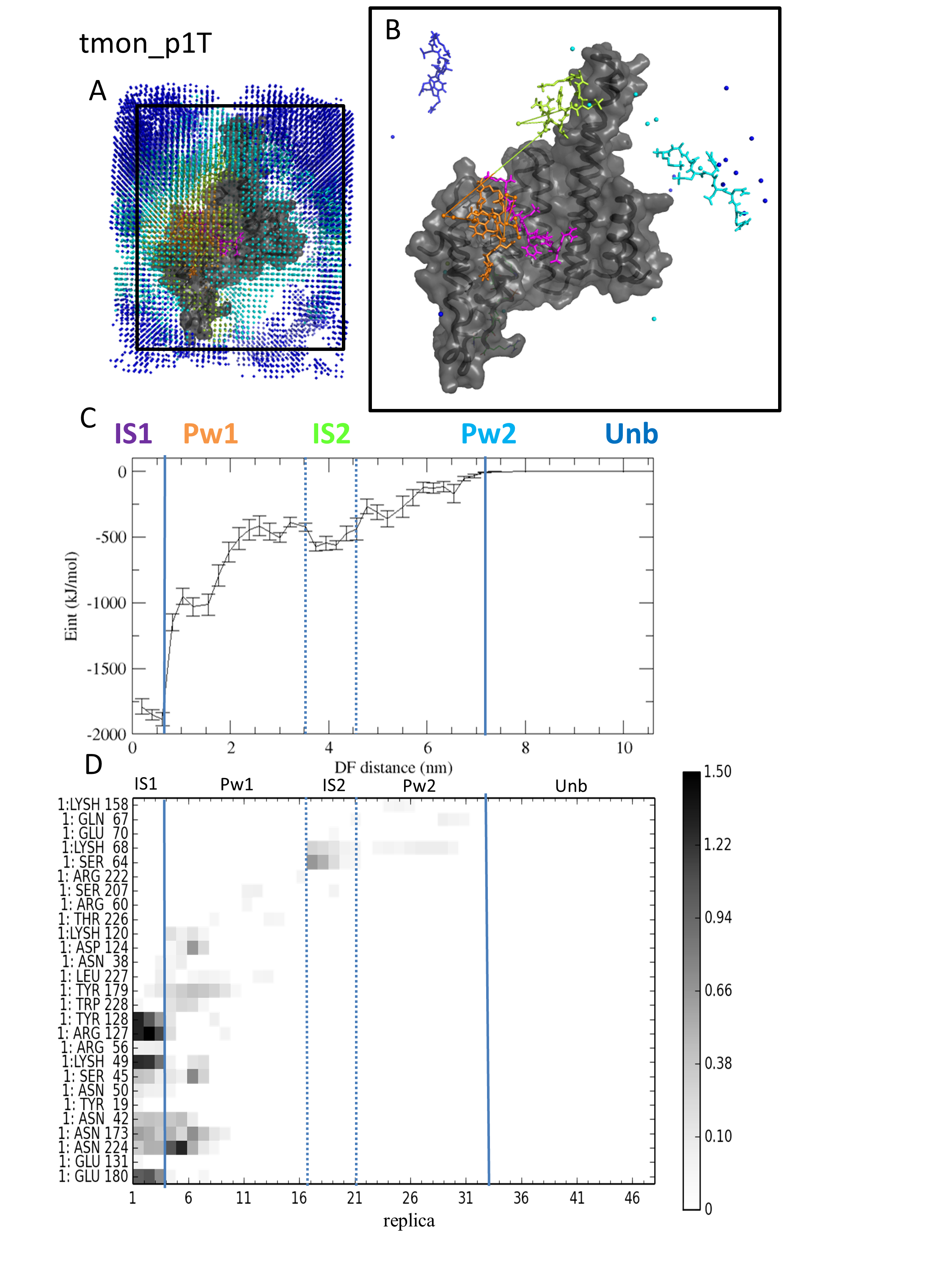

Supplement: S3 Fig — A) The volume sampled by the p1t phosphopeptide during the simulation is shown by dots around the 14-3-3ζ protein coloured based on their position along the pathway. The most probable points in space to find the peptide in for each replica (probability density peaks) are represented by larger spheres. B) Density peaks and a few representative structures corresponding to the density peaks, coloured according to their position along the pathway. Replica density peaks are connected by lines to visualize the binding pathway. The 14-3-3ζ protein in panels A-B is shown as a surface representation, with the two monomers shown in light and dark grey. C) Average interaction energy between 14-3-3ζ and the p1t. D) Interaction map between any atom of the pulled p1t peptide and the amino acids of the protein, summarized for each replica (only amino acids with at least 0.1 hydrogen bond/salt bridge on average are shown). The scale indicates the number of interactions. (TIF) [file pone.0180633.s006.TIF]

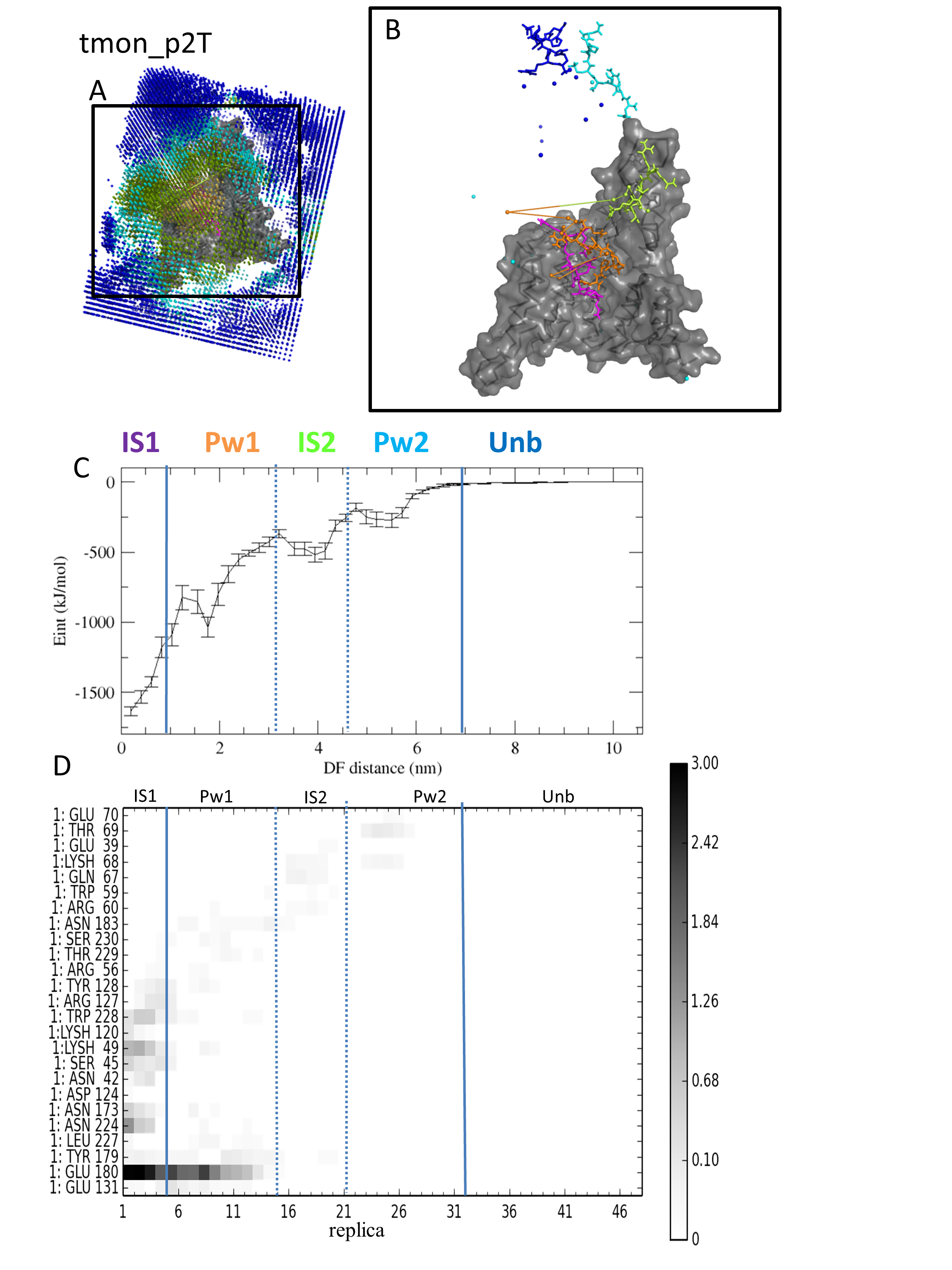

Supplement: S4 Fig — A) The volume sampled by the p2t phosphopeptide during the simulation is shown by dots around the 14-3-3ζ protein coloured based on their position along the pathway. The most probable points in space to find the peptide in for each replica (probability density peaks) are represented by larger spheres. B) Density peaks and a few representative structures corresponding to the density peaks, coloured according to their position along the pathway. Replica density peaks are connected by lines to visualize the binding pathway. The 14-3-3ζ protein in panels A-B is shown as a surface representation, with the two monomers shown in light and dark grey. C) Average interaction energy between 14-3-3ζ and the p2t. D) Interaction map between any atom of the pulled p2t peptide and the amino acids of the protein, summarized for each replica (only amino acids with at least 0.1 hydrogen bond/salt bridge on average are shown). The scale indicates the number of interactions. (TIF) [file pone.0180633.s007.TIF]

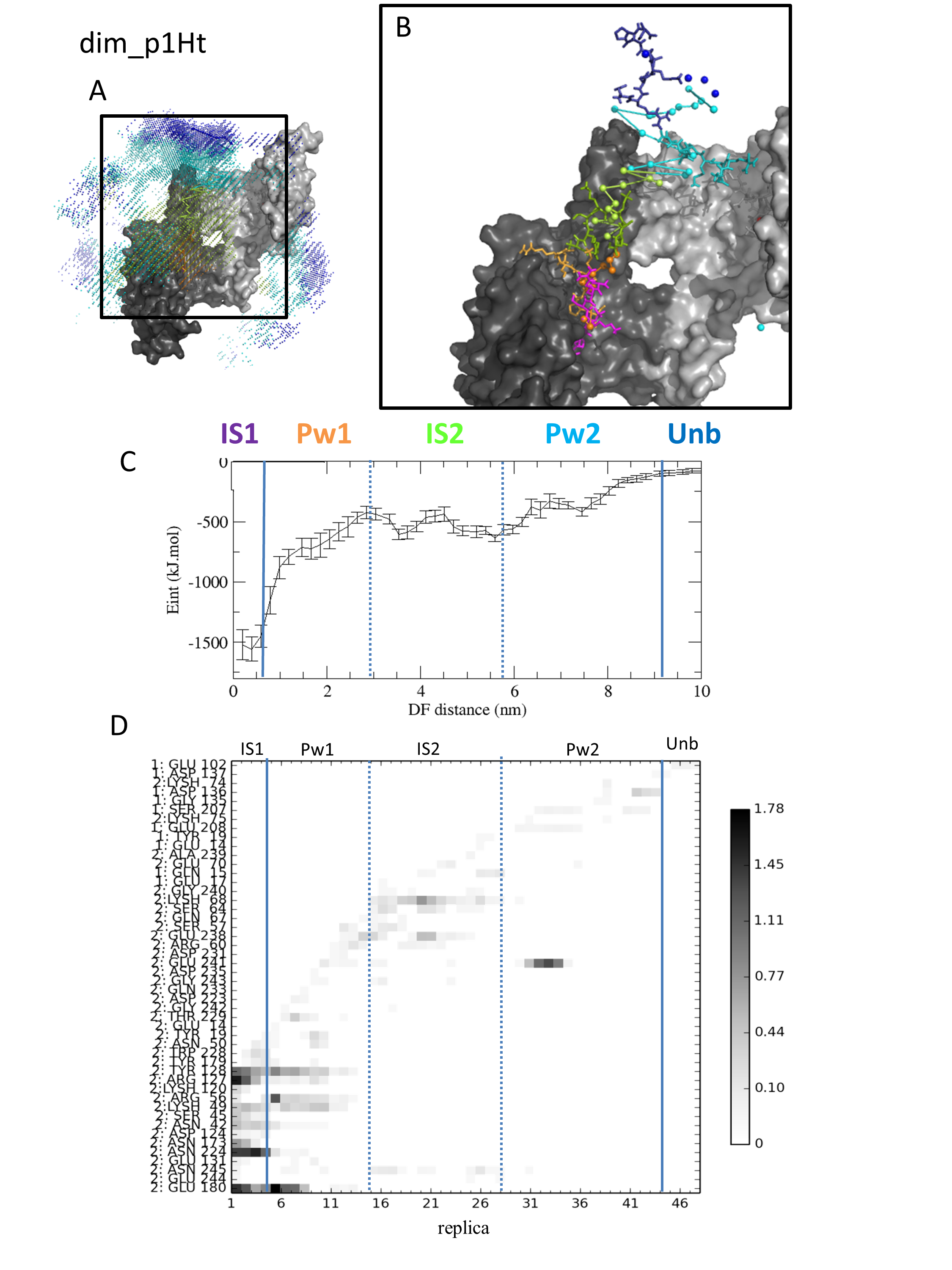

Supplement: S5 Fig — A) The volume sampled by the p1h phosphopeptide during the simulation is shown by dots around the 14-3-3ζ protein coloured based on their position along the pathway. The most probable points in space to find the peptide in for each replica (probability density peaks) are represented by larger spheres. B) Density peaks and a few representative structures corresponding to the density peaks, coloured according to their position along the pathway. Replica density peaks are connected by lines to visualize the binding pathway. The 14-3-3ζ protein in panels A-B is shown as a surface representation, with the two monomers shown in light and dark grey. C) Average interaction energy between 14-3-3ζ and the p1h. D) Interaction map between any atom of the pulled p1h peptide and the amino acids of the protein, summarized for each replica (only amino acids with at least 0.1 hydrogen bond/salt bridge on average are shown). The scale indicates the number of interactions. (TIF) [file pone.0180633.s008.TIF]

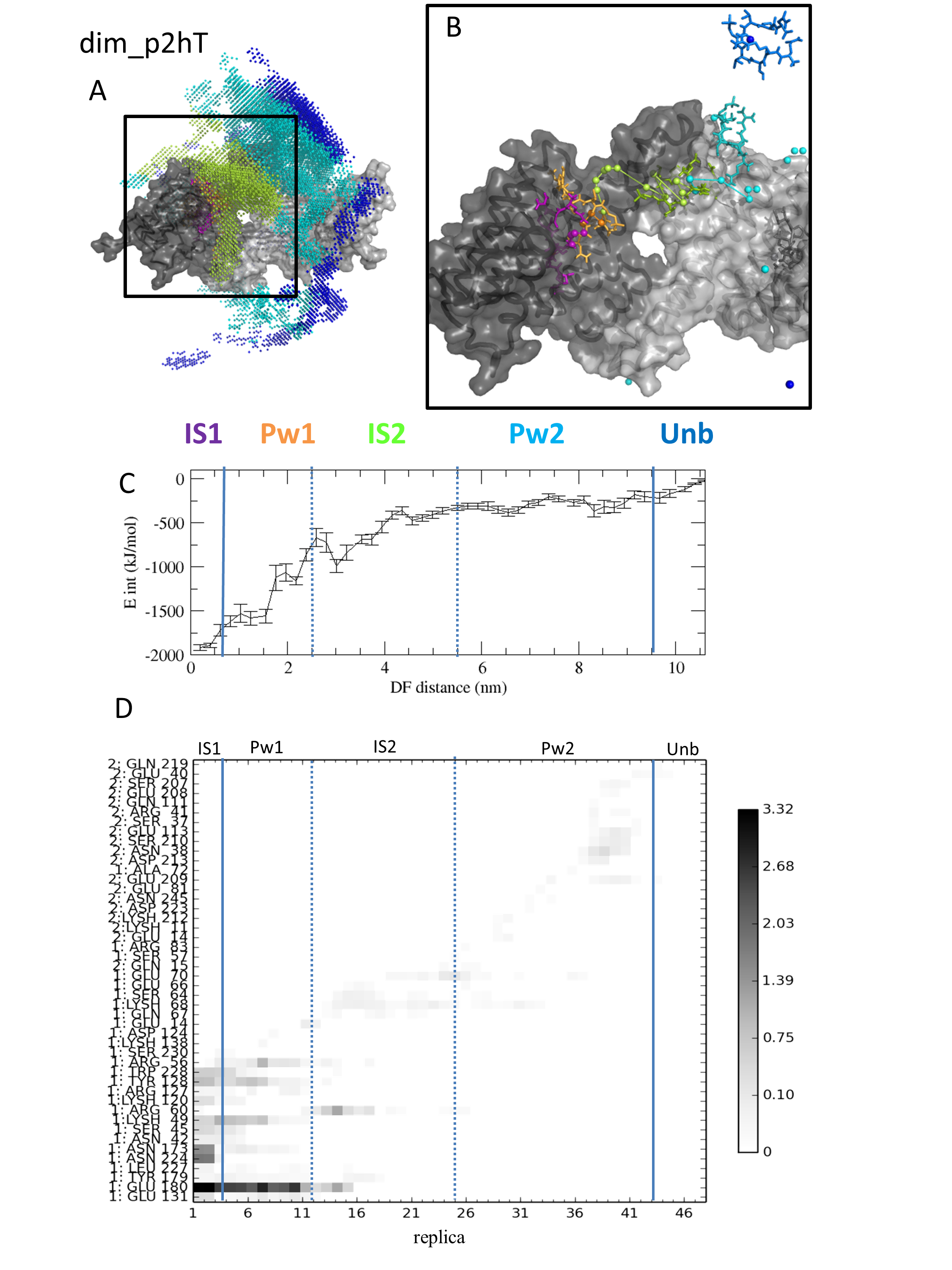

Supplement: S6 Fig — A) The volume sampled by the p2t phosphopeptide during the simulation is shown by dots around the 14-3-3ζ protein coloured based on their position along the pathway. The most probable points in space to find the peptide in for each replica (probability density peaks) are represented by larger spheres. B) Density peaks and a few representative structures corresponding to the density peaks, coloured according to their position along the pathway. Replica density peaks are connected by lines to visualize the binding pathway. The 14-3-3ζ protein in panels A-B is shown as a surface representation, with the two monomers shown in light and dark grey. C) Average interaction energy between 14-3-3ζ and the p2t. D) Interaction map between any atom of the pulled p2t peptide and the amino acids of the protein, summarized for each replica (only amino acids with at least 0.1 hydrogen bond/salt bridge on average are shown). The scale indicates the number of interactions. (TIF) [file pone.0180633.s009.TIF]

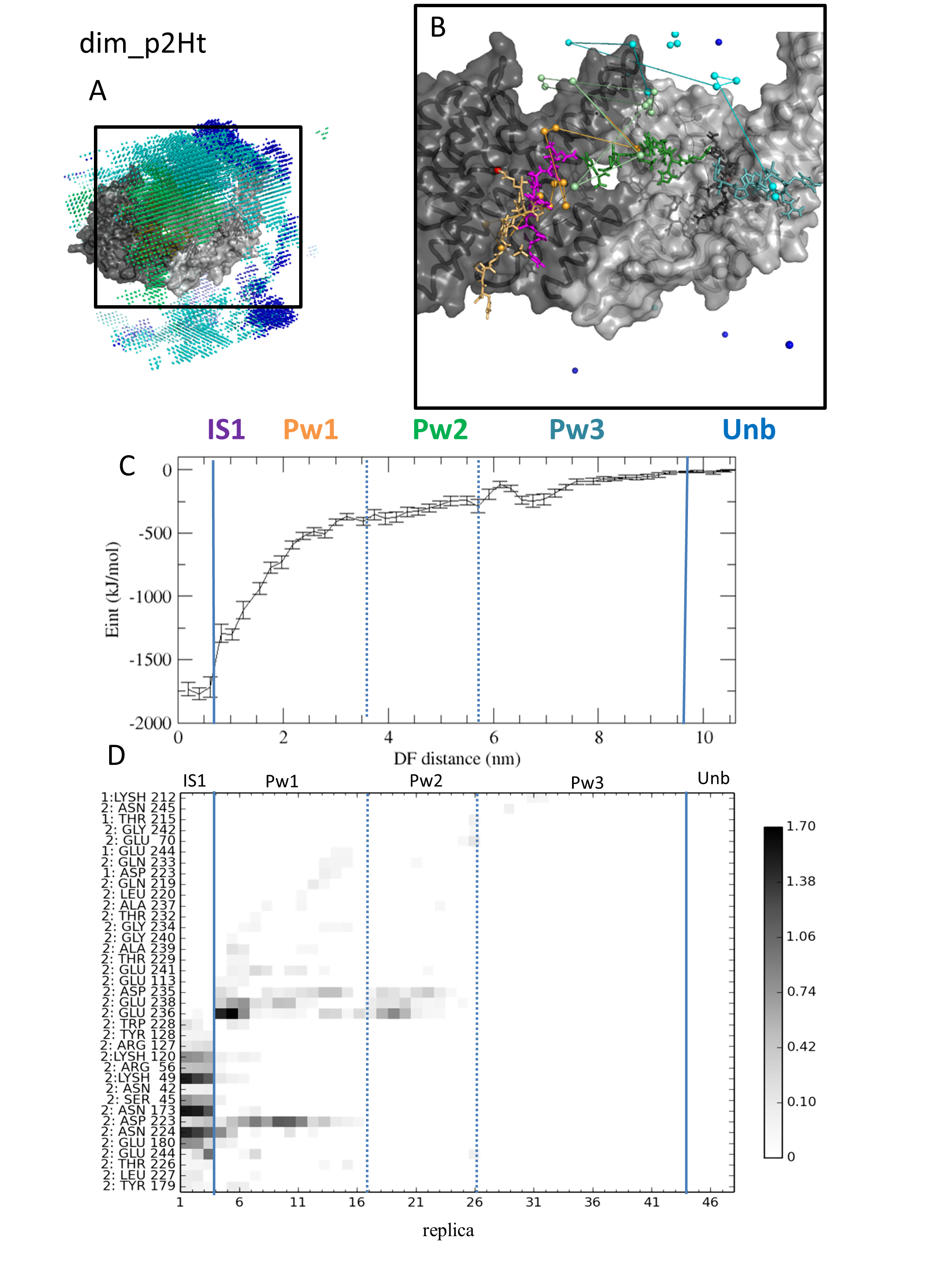

Supplement: S7 Fig — A) The volume sampled by the p2h phosphopeptide during the simulation is shown by dots around the 14-3-3ζ protein coloured based on their position along the pathway. The most probable points in space to find the peptide in for each replica (probability density peaks) are represented by larger spheres. B) Density peaks and a few representative structures corresponding to the density peaks, coloured according to their position along the pathway. Replica density peaks are connected by lines to visualize the binding pathway. The 14-3-3ζ protein in panels A-B is shown as a surface representation, with the two monomers shown in light and dark grey. C) Average interaction energy between 14-3-3ζ and the p2h. D) Interaction map between any atom of the pulled p2h peptide and the amino acids of the protein, summarized for each replica (only amino acids with at least 0.1 hydrogen bond/salt bridge on average are shown). The scale indicates the number of interactions. Note that dim_p2Ht did not follow the general pathway observed for other DF/HRE-MD simulations, and IS2 residues were not significant interaction partners. (TIF) [file pone.0180633.s010.TIF]

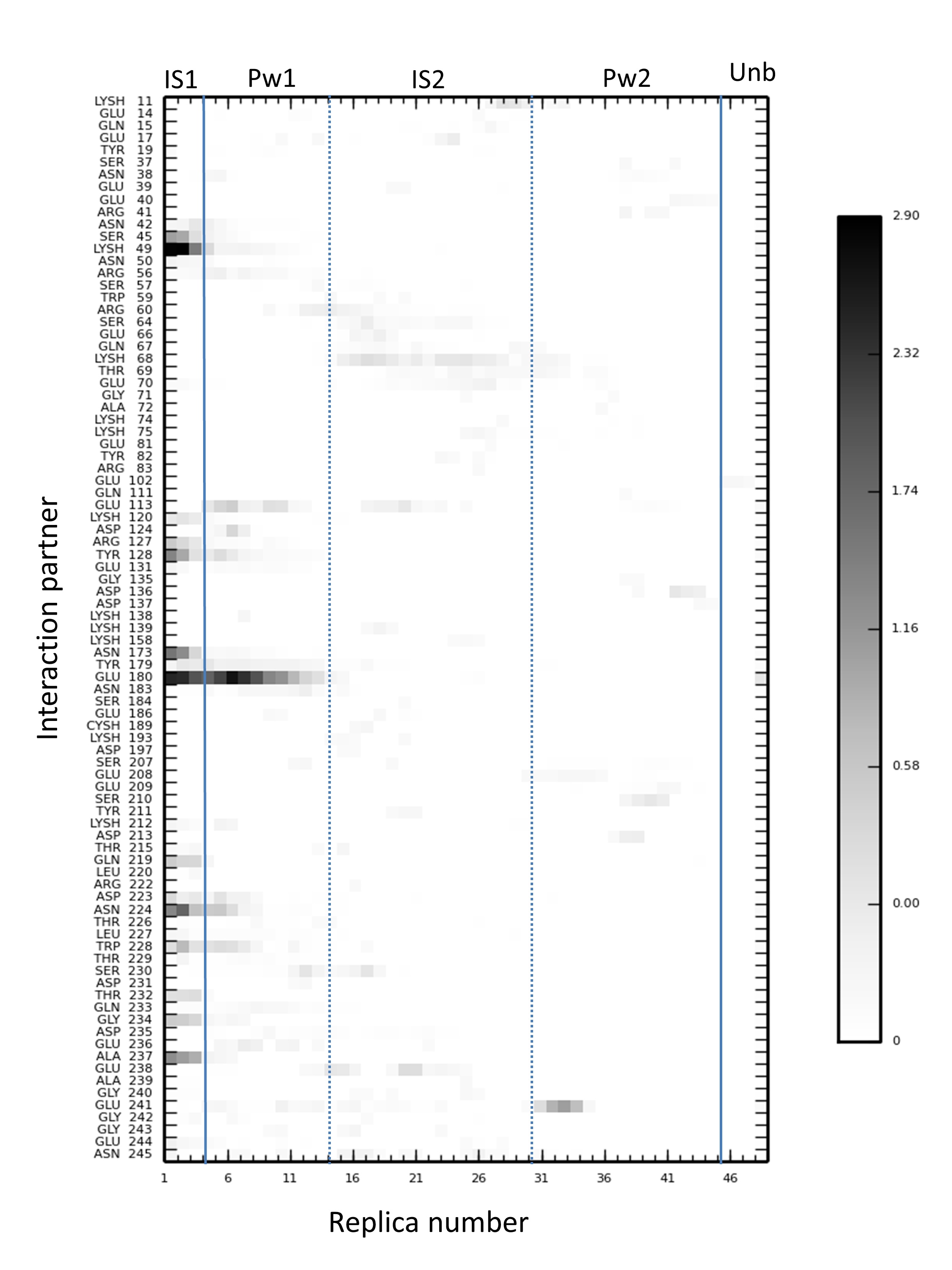

Supplement: S8 Fig — The map shows an average over the 8 HRE-MD simulations, where the average number of hydrogen bonds observed during the simulations are shown as the function of replica number. Replica 1–3 refers to the bound state in interaction site1 (IS1), and larger replica numbers correspond to higher DF distance from IS1. (TIF) [file pone.0180633.s011.TIF]

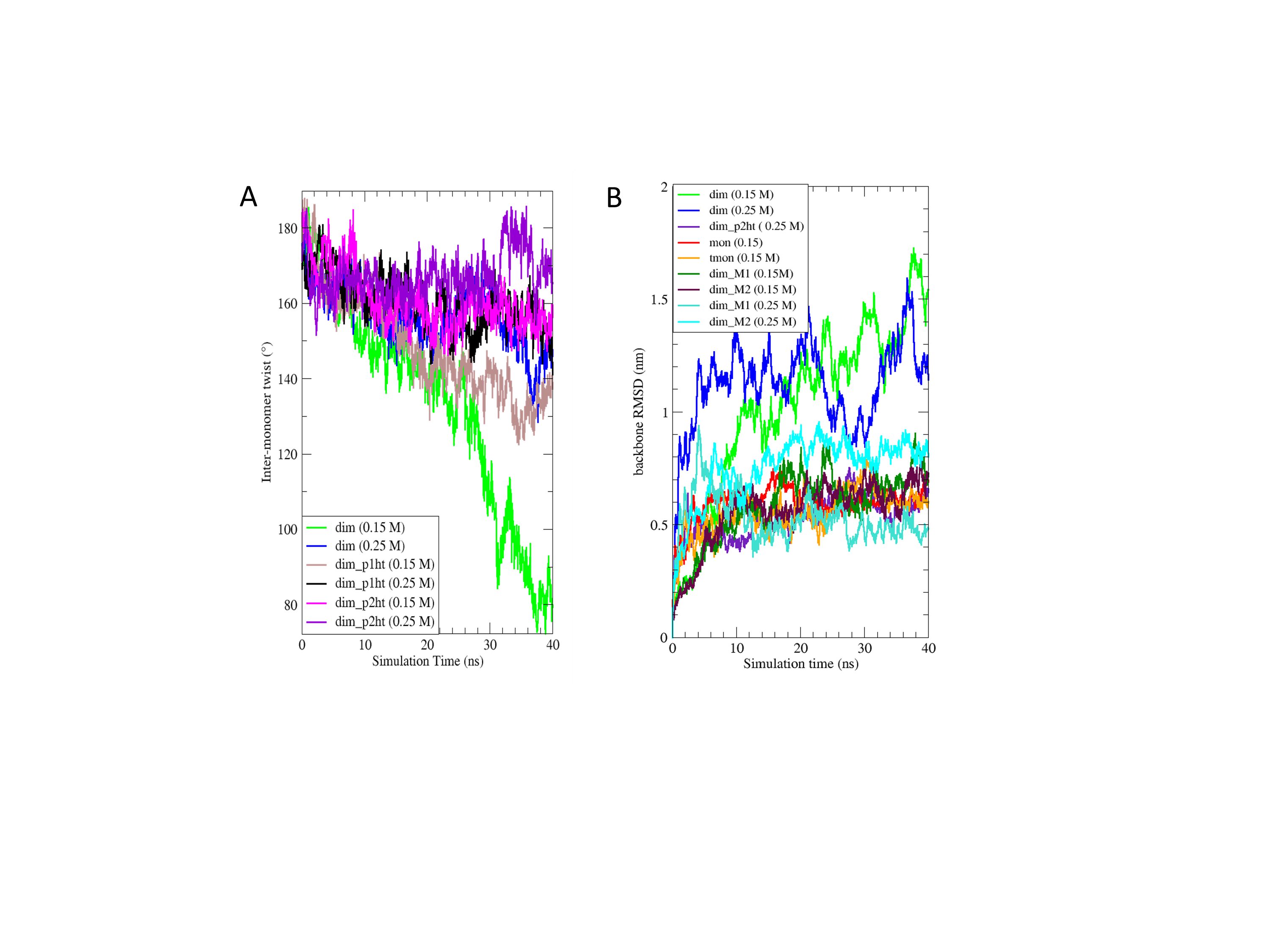

Supplement: S9 Fig — A) The drift of the monomer twist angle during MD simulations. B) Atom-positional root-mean-square deviation (RMSD) from the original conformation (derived from the crystal structure) during the various simulations. The structural deviation calculated for the individual monomers of simulation X are marked as X_M1 and X_M2, respectively. (TIF) [file pone.0180633.s012.TIF]

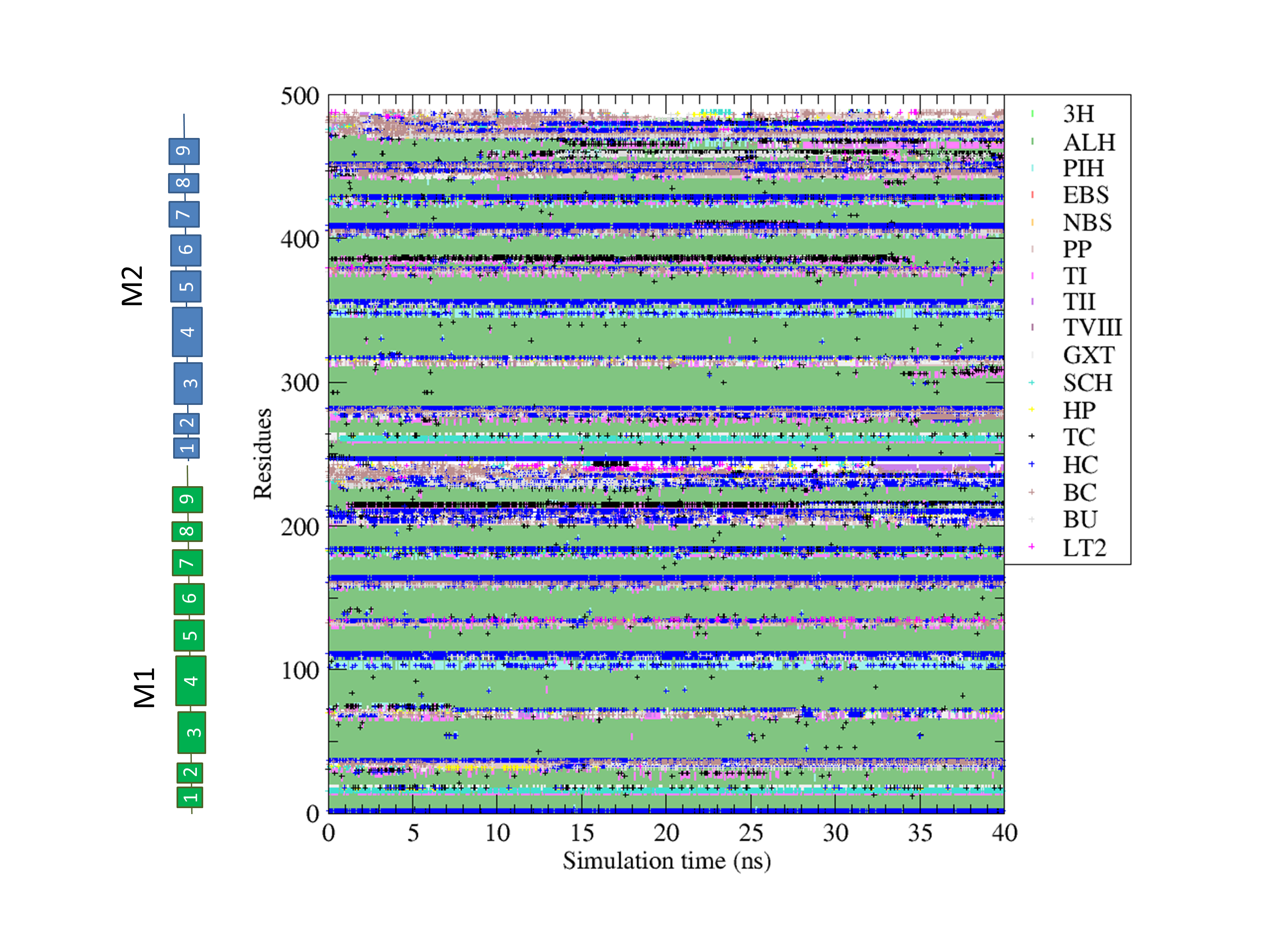

Supplement: S10 Fig — DISICL backbone analysis shows a stable secondary structure during the MD simulation dim. The left side of the Figure shows a schematic representation of the 14-3-3ζ helices. The most populated DISICL classes are depicted in the following colours: α-helix (ALH)–green, π-helix (PIH)–cyan, helix-cap (HC)–blue, turn-cap (TC)–black, polyproline-like (PP)–brown, turn type I (TI)–magenta, Turn type II (TII)–purple. For a description of abbreviations of DISICL secondary structure elements see S1 Table. (TIF) [file pone.0180633.s013.TIF]

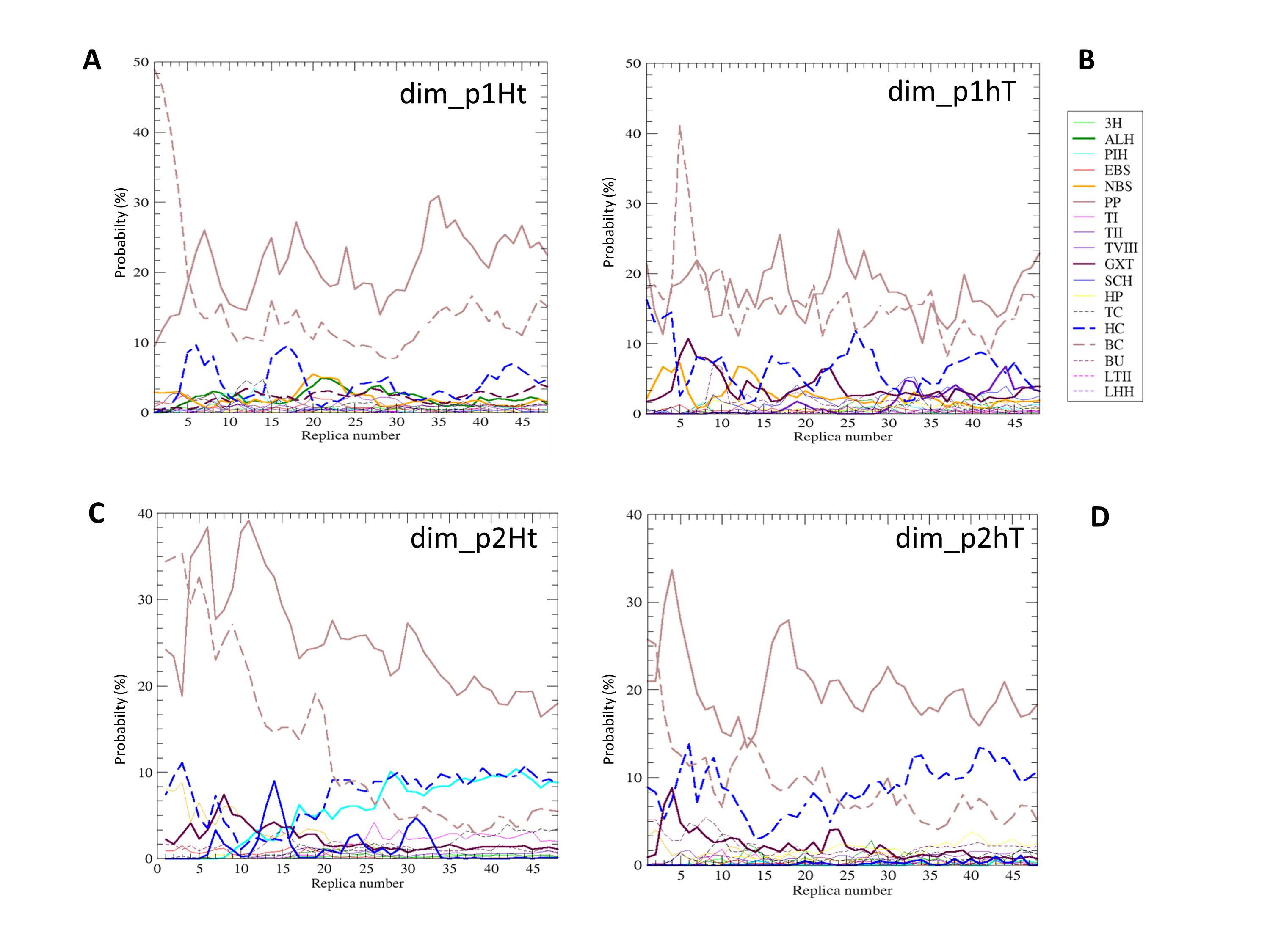

Supplement: S11 Fig — The population of secondary structure elements is dependent on the replica ID (and DF distance) within the dim DF/HRE-MD simulations. The change of the backbone secondary structure content was analysed by the DISICL algorithm. For a description of abbreviations of DISICL secondary structure elements see S1 Table. (TIF) [file pone.0180633.s014.TIF]

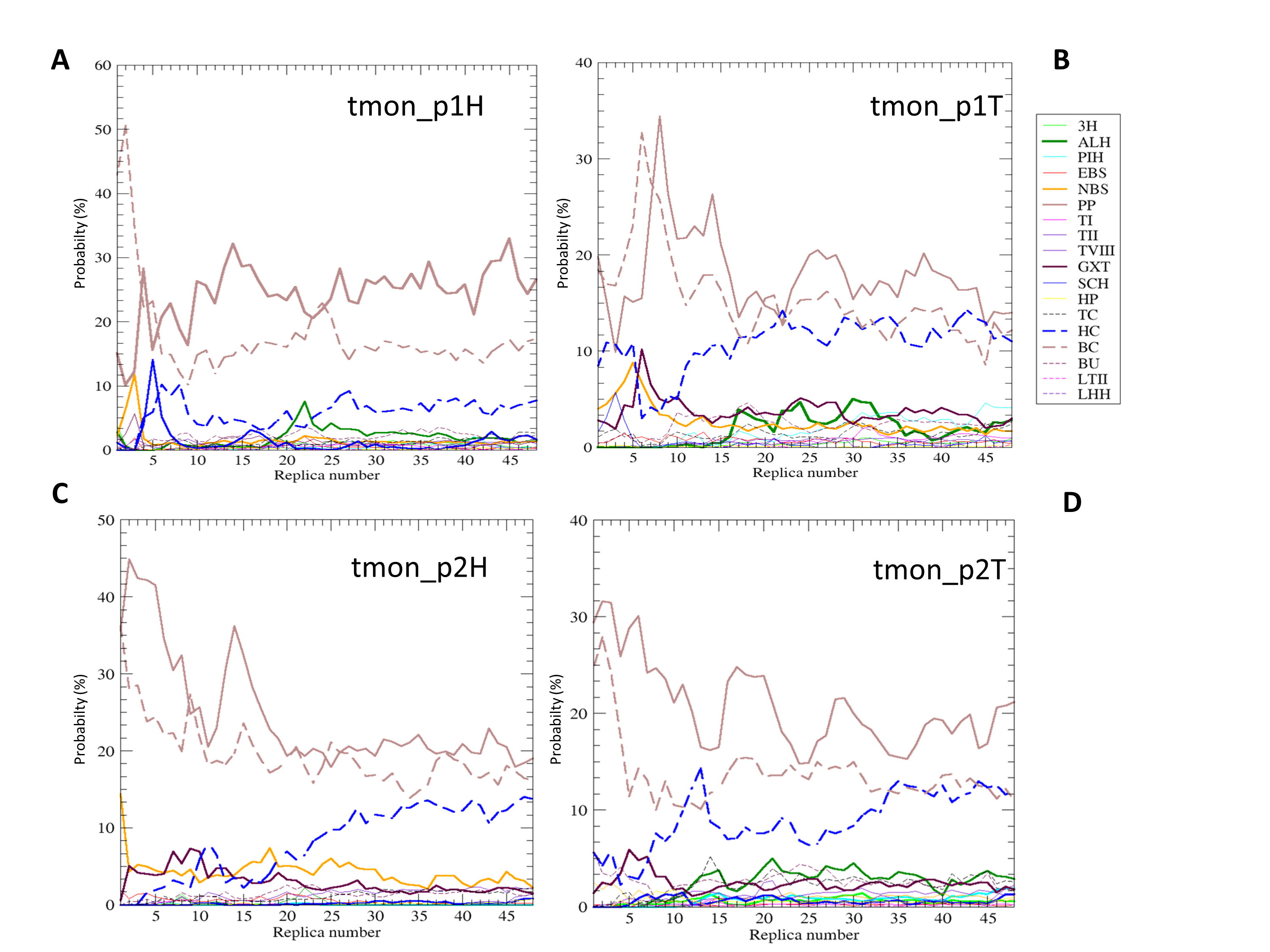

Supplement: S12 Fig — The population of secondary structure elements is dependent on the replica ID (and DF distance) within the tmon DF/HRE-MD simulations. The change of the backbone secondary structure content was analysed by the DISICL algorithm. For a description of abbreviations of DISICL secondary structure elements see S1 Table. (TIF) [file pone.0180633.s015.TIF]

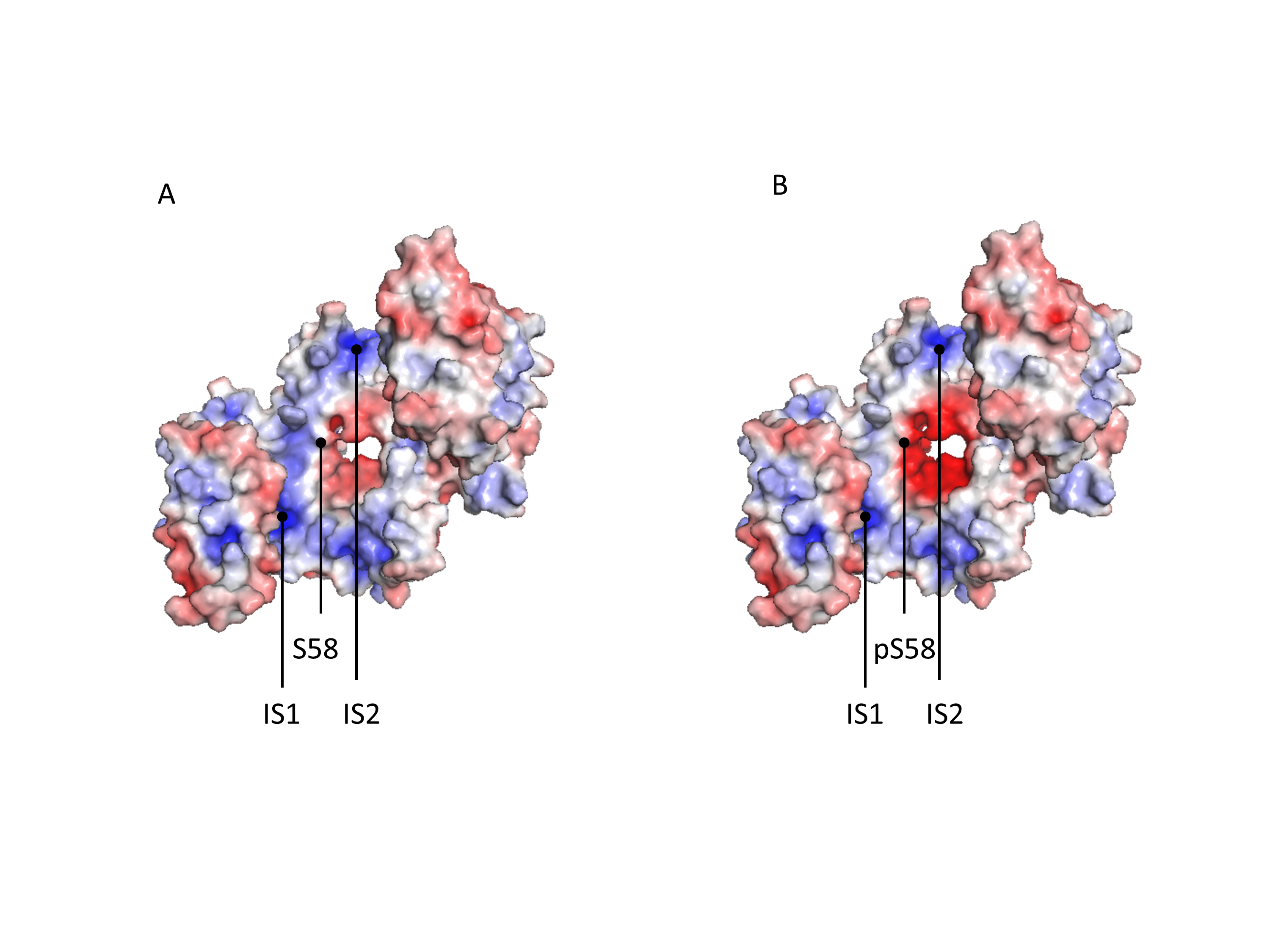

Supplement: S13 Fig — The IS1 and IS2 in A) 14-3-3ζ WT are connected by a mildly positive surface potential patch in contrary to B) 14-3-3ζ phosphorylated at S58, where the connection is disrupted by the negatively charged phosphoserine. Blue, white and red colour represents the positive, neutral, and negative surface patches, respectively. For the clarity, the 14-3-3ζ C-terminal tails are not shown. (TIF) [file pone.0180633.s016.TIF]

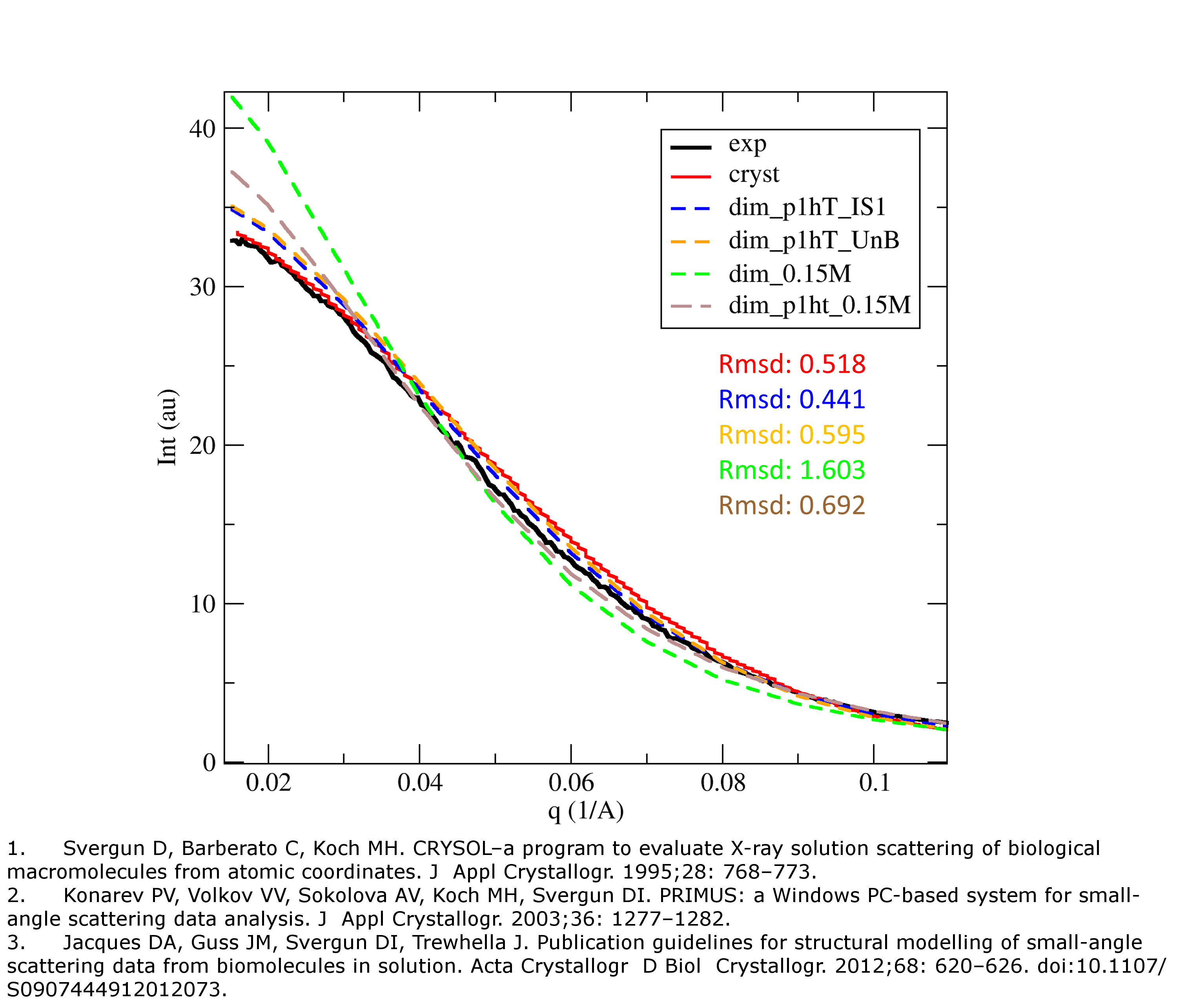

Supplement: S14 Fig — Small angle X-ray scattering calculated from the crystal structure (cryst, pdb code 2WHO), the IS1 and UnB stages dim_p1hT, and the unrestrained MD simulations with a low intermonomer twist angles (dim_0.15 dim_p1ht_0.15). The_SAXS curves were calculated using the software Crysol (1), and compared to the experimental SAXS curve (exp) after ensemble averaging. The full length 14-3-3ζ protein sample used for SAXS measurements was expressed and purified as described in Hritz et al.[4]. SAXS data were collected on the beamline BM29 BioSAXS ESFR in Grenoble, France. The concentrations of the 14-3-3-WT during the measurement were 1.18; 2.35 and 4.70 mg/ml in the 50 mM Tris buffer, pH = 8.0. The data were recorded at 20.12°C using the pixel 1M PILATUS detector at a sample-detector distance of 2.867 m, and a wavelength (λ) of 0.099 nm, covering the range of momentum transfer 0.025 nm−1 < s < 5 nm−1 (s = 4π sin(θ)/λ, where 2θ is the scattering angle). No radiation damage was observed during the data collection.The data were processed using standard procedures with the PRIMUS software (2). Solvent contributions (buffer backgrounds collected before and after the protein sample) were averaged and subtracted from the associated protein sample. A slight concentration dependency was noticeable. Therefore, the scattering curves collected at different concentrations were used to obtain a final zero concentration scattering curve through extrapolation according to the guidelines provided by (3). (TIF) [file pone.0180633.s017.tif]
